# Self-Assembly, Bioactivity, and Nanomaterials Applications of Peptide Conjugates with Bulky Aromatic Terminal Groups

**DOI:** 10.1021/acsabm.2c01041

**Published:** 2023-02-03

**Authors:** Ian W. Hamley

**Affiliations:** Department of Chemistry, University of Reading, Whiteknights, Reading RG6 6AD, United Kingdom

**Keywords:** Peptides, Peptide Conjugates, Self-Assembly, Bioactivity, Nanomaterials

## Abstract

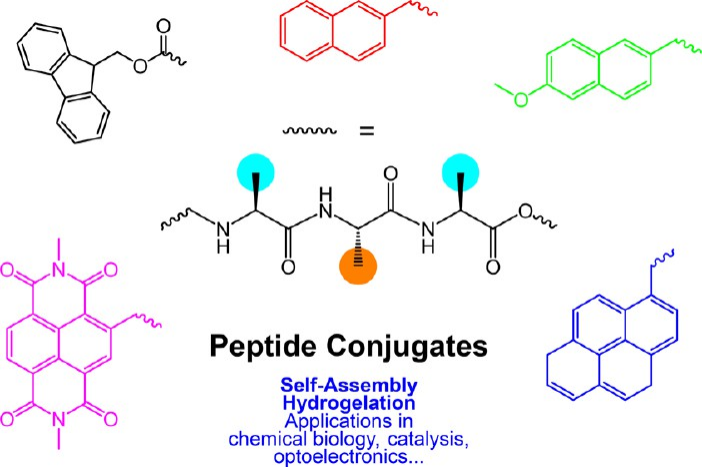

The self-assembly and structural and functional properties of peptide conjugates containing bulky terminal aromatic substituents are reviewed with a particular focus on bioactivity. Terminal moieties include Fmoc [fluorenylmethyloxycarbonyl], naphthalene, pyrene, naproxen, diimides of naphthalene or pyrene, and others. These provide a driving force for self-assembly due to π-stacking and hydrophobic interactions, in addition to the hydrogen bonding, electrostatic, and other forces between short peptides. The balance of these interactions leads to a propensity to self-assembly, even for conjugates to single amino acids. The hybrid molecules often form hydrogels built from a network of β-sheet fibrils. The properties of these as biomaterials to support cell culture, or in the development of molecules that can assemble in cells (in response to cellular enzymes, or otherwise) with a range of fascinating bioactivities such as anticancer or antimicrobial activity, are highlighted. In addition, applications of hydrogels as slow-release drug delivery systems and in catalysis and other applications are discussed. The aromatic nature of the substituents also provides a diversity of interesting optoelectronic properties that have been demonstrated in the literature, and an overview of this is also provided. Also discussed are coassembly and enzyme-instructed self-assembly which enable precise tuning and (stimulus-responsive) functionalization of peptide nanostructures.

## 1 Introduction

**Figure 1:**
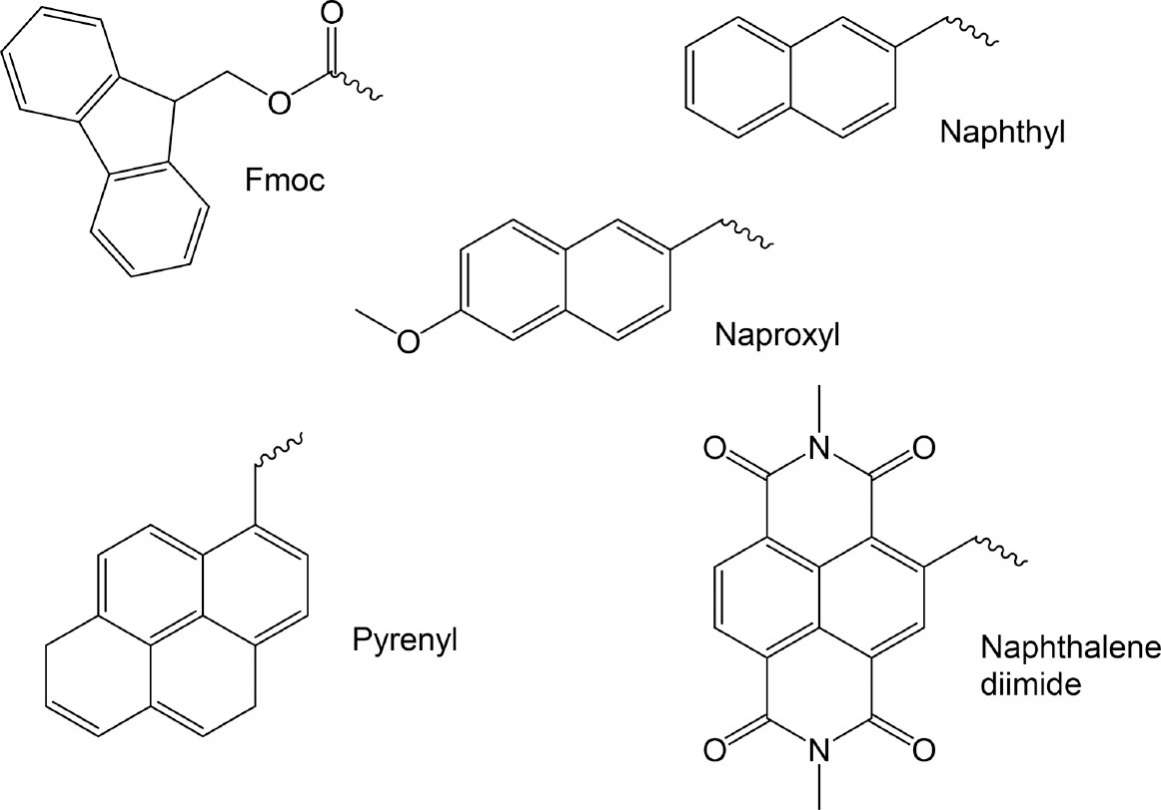
Summary of main types of bulky aromatic terminal groups discussed in this review. Different linker positions (and chemistries) to peptide chains to those shown have been used, as discussed herein.

This review is focused on peptide conjugates with bulky aromatic termini, including Fmoc [fluorenylmethyloxycarbonyl], naphthalene, pyrene, naproxen, aromatic diimides, and others. Examples are shown in Figure 1.

The Fmoc group, of course, is used as a protecting group in peptide synthesis;^1−3^ however, conjugates in which the Fmoc group is still attached have interesting aggregation properties which were among the first to be examined. Later, other bulky substituents such as naphthyl, pyrenyl and others were specifically conjugated to peptides to drive self-assembly through π-stacking interactions. These groups are typically attached for convenience at the N-terminus.

Reviews on peptide systems including N-terminal conjugates are available that cover conjugates with bulky N-terminal groups.^4−9^ Enzyme-driven self-assembly has also been reviewed^10,11^ as has the self-assembly of peptide conjugates in the cellular environment.^12,13^

Peptide conjugates comprising a bulky hydrophobic terminal motif and a relatively hydrophilic peptide will be amphiphilic, and the use of bulky aromatic units means that π–π stacking interactions are important molecular aggregation processes. Noncovalent interactions between the π orbitals of aromatic rings lead to π–π stacking, also known as π-stacking, with several configurations of rings including face–face (with or without displacement) or edge-to-face interactions between the rings. The important role of π-stacking among aromatic residues is recognized for amyloid fibril formation.^14^ Addition of non-natural bulky aromatic residues generally enhances self-assembly into extended structures, as highlighted in numerous examples discussed herein. Such structures thus represent a valuable handle to drive peptide self-assembly. This can also lead to novel properties and activities, as highlighted throughout this Review.

Peptide conjugate molecules have a tendency to self-assemble and form hydrogels with a fibrillar structure that entraps water within its mesh structure. A number of methods have been developed to produce hydrogels of peptide conjugates, including pH switching, solvent switching, heating/cooling and enzymatic methods, i.e., treatment of nonaggregating precursors with enzymes that remove units that hinder aggregation (e.g., dephosphorylation) or facilitate the coupling of nonaggregating precursors. Examples of all of these methods are discussed herein and in a previous overview of hydrogel preparation methods.^15^ It is possible to tune the rheological properties (shear modulus) over a considerable range by judicious choice of peptide conjugate, concentration and other variables as highlighted by numerous examples discussed below. This has an impact on properties such as cytocompatibility and the facilitation of 2D or 3D cell culturing. Peptide conjugate structures have a remarkable diversity of other functions including optoelectronic properties, and uses slow-release delivery systems, supports for catalysis and others, as highlighted in the many examples discussed herein.

This review does not include the extensive literature on peptides terminally modified with lipid chains (lipopeptides or peptide amphiphiles), a topic that has been extensively reviewed elsewhere.^16−23^ As is characteristic of overviews on fast-moving topics with broad interest, the current review provides a perspective of selected interesting works, since it is not possible to cover every paper on the subject. Also this field does not have rigid boundaries. For example, the term “bulky” terminal group does not have a rigorous definition, and the focus here is mainly on N-terminal conjugates since the majority of research has been on such hybrids.

In the following, the single letter abbreviation of amino acids is used and d-amino acid residues are indicated with lower case letters. Also, for brevity notation such as Nap-FF is used for a naphthalene conjugate to diphenylalanine, without specifying the particular naphthyl-peptide linking position or linker group.

## 2 Fluorenylmethyloxycarbonyl

### 2.1 Fmoc-FF and Related Fmoc-Dipeptides and Mixtures

**Figure 2:**
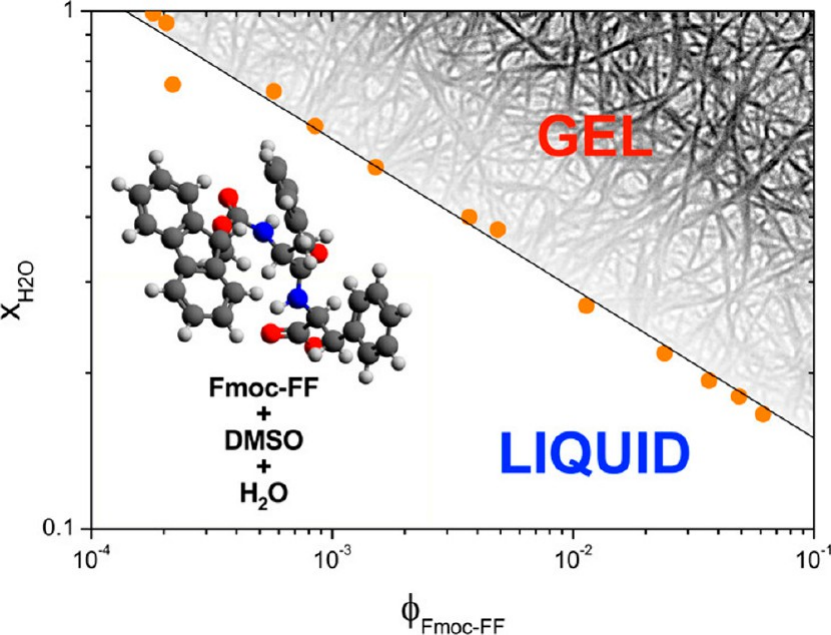
Gel phase diagram for Fmoc-FF as a function of water mole fraction in DMSO/H_2_O mixed solvent. Reproduced from ref (26). Copyright 2014 American Chemical Society.

Fmoc-diphenylalanine (Fmoc-FF) is probably the most widely studied N-terminally modified peptide. Fmoc-FF forms stiff hydrogels with a fibrillar nanostructure.^24,25^ The hydrogel is able to support live CHO (Chinese hamster ovary)^24^ cells, and it can support 2D or 3D culture of bovine chondrocyte^25^ cells. Hydrogels can be formed by dilution of a HFIP stock solution with water at sufficiently high concentration, as reported by Gazit’s group,^24^ or by pH switch (initial increase with NaOH, then acidification by dropwise addition of HCl).^25^ As a hydrophobic molecule, Fmoc-FF can be initially dissolved in DMSO (or HFIP) and upon dilution with water, hydrogelation is observed at concentrations as low as 0.01 wt %.^26^ A gel phase diagram for Fmoc-FF has been presented (Figure 2) along with detailed rheological characterization of selected gels.^26^

The different preparation methods and high sensitivity to preparation conditions lead to hydrogels with a very large variation in reported rheological properties (modulus).^27^ The preparation of gels from organic solvent followed by addition of water leads to gels with a modulus that can be controlled by the choice of starting organic solvent.^28^ This was shown to be related to changes in fibril morphology. Metastable gels are formed in acetone in which crystals are observed and these were used to obtain samples allowing single crystal structure determination from X-ray diffraction (XRD).^28^ The final pH of the gels is the principal determinant of the mechanical properties independent of the method of gel formation. A slow pH reduction method was also presented that is based on hydrolysis of glucono-δ-lactone (GdL), which gives more reproducible gel properties.^27^ A model for the packing of Fmoc-FF in fibrils was proposed based on interlocked stacking of β-sheets driven by π-stacking interactions, leading to a hollow core structure, i.e., a nanotube-like structure.^29^ This would give rise to a distinct form factor in small-angle scattering data, which seems worthy of further examination. The aggregation of Fmoc conjugates can lead to significant shifts in p*K*_a_, as exemplified by acid titration studies on Fmoc-FF-OH for which two apparent p*K*_a_ values are observed, both significantly different from the expected p*K*_a_ = 3.5.^30^ This was shown to be correlated with pH-dependent morphological differences, specifically flexible fibrils forming a hydrogel were observed at high pH, but flat ribbons were noted at intermediate pH values in nongeling solutions. Shifts in p*K*_a_ were likewise reported for Fmoc-FG, Fmoc-GG, and Fmoc-GF, and the apparent p*K*_a_ values were found to correlate with the hydrophobicity of the Fmoc-dipeptides.^31^ Fmoc-GG and Fmoc-FG only self-assemble below their apparent p*K*_a_ (i.e., in their protonated form), in contrast to Fmoc-FG which self-assembles above and below its apparent p*K*_a_. It was also shown to exhibit the unusual property of gelation on heating.^31^

Fmoc-FF hydrogels comprise a network of peptide nanotube-like fibrils and exhibit interesting optical properties resulting from quantum confinement, i.e., creation of excitons.^32^ Photoluminescence excitation is observed, the development of which was monitored during hydrogel self-assembly. Metallogels have been produced by self-assembly of Fmoc-FF in the presence of various metal ions, and the conformational and morphological behavior were examined using an arsenal of spectroscopic and microscopic techniques.^33^ However, certain ions favor the formation of structures other than β-sheets, i.e., “superhelices” or random coils, depending on the mixing ratio of Fmoc-FF and metal ion. The metallogels prepared with Na^+^ or Zn^2+^ are able to bind DNA, the latter particularly rapidly.^33^

Hydrogels containing mixtures of Fmoc-FF with Fmoc-R have been prepared due to interest in the affinity of arginine to hydroxyapatide.^34^ The hydrogels supported adhesion of the fibroblasts studied. The chirality of fibrils containing Fmoc-FF can be tuned by coassembling with achiral pyridine derivatives due to different stacking modes (H- or J-aggregates).^35^

The hydrogelation of Fmoc-dipeptide derivatives including Fmoc-AA, Fmoc-aa, Fmoc-GG, and others has been investigated and critical gel concentrations were determined as well as pH and temperature conditions for gel stability.^36^ Fibrillar structures were imaged by electron microscopy. Fmoc-AA forms fibrils, although detailed computer simulations indicate that the dialanine adopts a mainly PPII structure, rather than β-sheets and that self-assembly is driven by Fmoc stacking rather than the typical hydrogen bonding of β-sheets.^37^ The hydrogelation of several Fmoc-dipeptides was also noted by Ulijn’s group and proliferation of chondrocytes was observed on Fmoc-FF as well as mixtures of this with Fmoc-GG or Fmoc-K.^25^ This group also used Fmoc-FF as a structural component of bioactive scaffolds for 3D cell culture, in mixtures with bioactive Fmoc-RGD containing the bioactive integrin adhesion motif^38^ RGD.^39^ Mixtures of Fmoc-FF with Fmoc-K, Fmoc-D or Fmoc-S were also investigated for 2D and 3D culturing of several types of cell. Fmoc-FF/Fmoc-S hydrogels were found to be compatible with all three types of cell examined and was the only system able to support 3D culture of chondrocytes.^40^ It was noted that it was not possible to disentangle the relative importance of mechanical properties and chemical functionalities on controlling cell behavior in Fmoc-peptide gels, although both are important and can be varied by formulation. The rheological properties of hydrogels formed by coassembly of Fmoc-FF with Fmoc-S, Fmoc-D, Fmoc-K, or Fmoc-Y have been measured and gel microparticles fabricated by the Pickering emulsion method have been examined as systems for catalysis using a model enzyme immobilized in the particles.^41^

As mentioned above, the properties of Fmoc-FF hydrogel mixtures are sensitive to preparation method, as exemplified by a study on the mechanical properties and morphology of Fmoc-FF/Fmoc-GG mixtures.^42^ Such variable properties motivated the use of GdL hydrolysis as a slow pH reduction process in the preparation of more homogeneous Fmoc-peptide hydrogels (shown for a range of Fmoc-dipeptides) with more reproducible properties.^43,44^ In a more recent development, the urease-catalyzed hydrolysis of urea has been used as a method to prepare homogeneous gels by slow pH increase (to pH 9), using for example Fmoc-K and related Fmoc-amino acids.^45^ The gels are visibly more homogeneous than those obtained by simple addition of NaOH and the shear modulus can also be increased by adjustment of the urease concentration. The kinetics of pH increase and gelation depend on the initial (acidic) pH conditions and the nature of the acid used to create the starting state.^45^ Fmoc-FpY [pY: phosphotyrosine] coassembles with Fmoc-S, Fmoc-T, or Fmoc-RGD when triggered by alkaline phosphatase (ALP) hydrolysis to form fibrils (and hydrogels), whereas the individual components form micelles.^46^

Hydrogelation has also been observed for Fmoc-LD, Fmoc-AD, and Fmoc-ID and the gels show high thermostability.^47^ Fmoc-LD hydrogels can be used to incorporate adamantanamine derivatives, which are inherently nonantigenic antiviral drugs The gels facilitate antigen presentation, as shown by the production of high titers of specific antibodies in a rabbit model.^47^ Thin hydrogel membranes can be fabricated using electrodeposition of peptide conjugates such as Fmoc-LG.^48^ Such layers can be used to seed the growth of fibrils from a solution of such a conjugate.^49^ Electrochemical methods (cyclic voltammetry or multiple pulse amperometry) can also be used to probe the surface characteristics of peptide conjugate hydrogels, such as determination of p*K*_a_ and measurement of charge and ion binding dynamics.^50^

Linking Fmoc-FF C-terminally to a gold complex, Au(I)-trisulfonated-triphenylphosphane leads to a peptide metalloamphiphile which self-assembles into luminescent micelles in aqueous buffer.^51^ Coassembly of Fmoc-FF with FF leads to a composite hydrogel in which FF crystallization is modulated compared to FF crystallization from solution.^82^ The kinetics of formation of the microcrystalline aggregates was also investigated.

Fmoc-dipeptides containing β-alanine (βA) have been developed since β-amino acids are resistant to proteolysis.^52^ Conjugates Fmoc-βA-L or Fmoc-βA-F form fibrillar hydrogels, and the former indeed shows minimal degradation in proteinase K. The hydrogels show sustained release of model drugs (vitamin B2 or B12).^52^ Fmoc-βAH (Fmoc-carnosine) also forms β-sheet fibrils above a critical aggregation concentration, and the chelation of zinc ions by the terminal H was examined.^53^ This also changed the fibril morphology, and hydrogelation was observed at a suitable concentration of Zn^2+^ ions. An Fmoc conjugate containing a β-amino acid, Fmoc-β-phenylalanine forms a hydrogel that undergoes syneresis, i.e., undergoes “self-shrinkage” with corresponding change in molecular packing (J- to H-aggregate type).^54^

### 2.2 Fmoc-Amino Acids

Conjugation to Fmoc remarkably leads to self-assembly of single amino acids. Hydrogelation (in Na_2_CO_3_) of Fmoc-L and Fmoc-K (and mixtures) was demonstrated.^55^ Several Fmoc-amino acids have anti-inflammatory properties,^56^ although Fmoc shows some cytotoxicity.^6^ Fmoc-F forms fibrillar hydrogels, as demonstrated in a paper that also presents other derivatives of F with bulky N-terminal substituents.^57^ Fmoc-F hydrogels have been used to host silver nanoclusters. Without requiring reducing agents, silver ions are complexed with the carboxylate groups of the free carboxylic acids of Fmoc-F and are reduced spontaneously in diffused sunlight to form silver nanoclusters within the gel,^58^ following a method also demonstrated for Fmoc-VD hydrogels.^59^ Fmoc-L is a Fmoc-amino acid with anti-inflammatory activity,^56^ and it forms hydrogels when mixed with Fmoc-K.^60^ Fmoc-amino acids can be used to create hydrogels for enzyme immobilization, as demonstrated with Fmoc-K/Fmoc-F mixtures with added enzymes including laccase, horseradish peroxidase, or α-chymotrypsin.^61^ Improved activity and stability of the hydrogel-immobilized enzymes was reported.

Nilsson’s group carefully examined the self-assembly and gelation properties of Fmoc-F-based compounds with halogenated phenyl groups.^62,63^ Distinct fibril morphologies, spectroscopic characteristics, and hydrogel rheological modulus are reported, depending on the nature of the halogen and position of substitution.^62^ In one comparison, Fmoc-F5-Phe (containing perfluorinated phenylalanine) was shown to gel much more rapidly and at lower concentrations than Fmoc-Y.^64^ Additional studies concerned coassembly of Fmoc-F5-Phe with Fmoc-F5-Phe-dEG (dEG: diethylene glycol)^65^ or of Fmoc-F5-Phe with Fmoc-Phe or monohalogenated Fmoc-Phe derivatives,^66^ or with Fmoc-FF.^67^ In the latter case, a focus on gel modulus revealed that a storage modulus near 200 kPa for a 1:1 mixture is attainable, corresponding to a highly rigid supramolecular hydrogel.^67^ Whereas Fmoc-l-DOPA(acetonated)-d-Phe-OMe and FF on their own form globular aggregates, coassembly leads to self-assembled nanostructures resembling red blood cells or spherical structures with bulges on their outer surface (like white blood cells), depending on concentration.^68^ The coassembly of Fmoc-l-DOPA(acetonated)f-OMe with other diphenylalanine analogues was also examined. A review focused on coassembly of peptide conjugates is available.^69^

Fmoc-F or Fmoc-Y gels prepared using GdL hydrolysis have demonstrated slow release of model dyes.^70^ Self-assembly of Fmoc-Y can be induced via ALP treatment of Fmoc-pY precursor, which itself forms micelles above a critical micelle concentration.^71,72^ The kinetics of the self-assembly and gelation process were probed using time-resolved rheology and spectroscopic techniques.^72^ The hydrogel modulus can be tuned via concentration of ALP.^73^ The modulation of nanostructure by substitution within Fmoc-F(4-X)F was examined, where F(4-X) denotes phenylalanine modified at the 4-position with electron withdrawing groups, e.g., F, CN, NO_2_.^74^ Dipeptides were formed via thermolysin-mediated condensation of Fmoc-F(4-X)-OH and F-NH_2_ (vide infra).

### 2.3 Other Fmoc-Peptides

**Figure 3:**
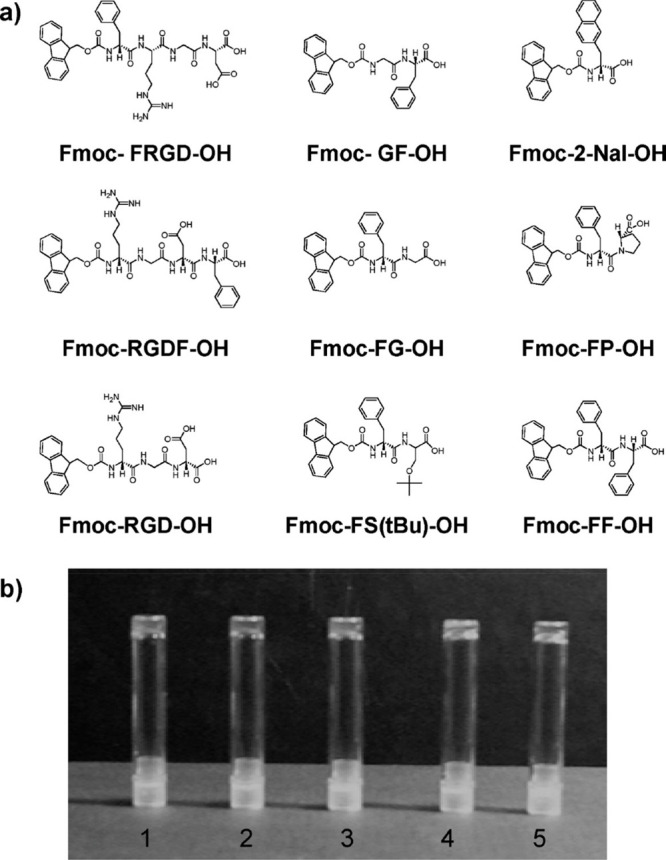
(a) Fmoc conjugates screened for hydrogelation. (b) Hydrogels formed by (1) Fmoc-FRGD; (2) Fmoc-RGDF; (3) Fmoc-2-Nal; (4) Fmoc-FG; (5) Fmoc-FF. Reproduced from ref (75). Copyright 2009 American Chemical Society.

Gazit and co-workers showed that other conjugates based on Fmoc-F (and a related naphthyl alanine 2-Nal derivative, and Fmoc-RGD) such as those shown in Figure 3 form hydrogels.^75^ The morphology was investigated using scanning electron microscopy and transmission electron microscopy (TEM) and viability of CHO cells was examined for a subset of the derivatives. Good viability was observed for Fmoc-FRGD and Fmoc-RGDF (after 1 day incubation), whereas the cells on Fmoc-2-Nal showed low viability.^75^ The rheological properties, structure (via fiber XRD) and appearance (turbidity) of these samples was also investigated.^76^

The self-assembly and hydrogelation properties were analyzed for Fmoc-VLK(Boc) and Fmoc-K(Boc)LV, both containing K protected by N^ε^-*tert*-butyloxycarbonate (Boc).^77^ The former peptide forms highly anisotropic fibrils in borate buffer which show local alignment, and also hydrogels with flow-aligning properties. In contrast, Fmoc-K(Boc)LV forms highly branched fibrils that produce isotropic hydrogels with a higher modulus. The distinct self-assembled structures were ascribed to conformational differences, as revealed by spectroscopic probes of secondary structure and X-ray diffraction.^77^ In related work, Fmoc-K(Fmoc) has been shown to form fibrillar gels in aqueous buffer solutions with distinct pH-dependent rheological properties.^78^ Fmoc-K(Fmoc)D, another conjugate with a double branched Fmoc structure, forms hydrogels with a very low critical gel concentration.^79^ The hydrogels are suitable for 2D or 3D cell culturing and hybrid gels of this conjugate with polyaniline (prepared by oxidative polymerization in situ) show metal-like electrical conductivity as well as DNA binding ability.^79^ Attaching NDI (naphthalene diimide, section 6) via a lysine side chain in Fmoc-KK(NDI) gives a conjugate that forms hydrogels with a morphology of nanotapes driven by π-stacking of both Fmoc and NDI units.^80^ Fmoc-KK(Pyrene)K meanwhile forms β-sheet nanotapes, a distinct morphology to that for peptides lacking the terminal N-terminal Fmoc.^81^

Other Fmoc-tripeptides studied include Fmoc-FWK (uncapped or C-terminal capped).^83^ The uncapped peptide self-assembles into helical structures such as twisted nanoribbons at pH 11.2–11.8, or nanofibers at pH 5 and 12 or flat ribbons composed of many fibrils for pH 6–11. However, only nanofibers were observed for Fmoc-FWK-NH_2_ across a range of pH.

The conformation and self-assembly of Fmoc-peptides bearing the integrin cell adhesion motif RGD (or scrambled sequence GRD) has been studied.^84^ It was shown that Fmoc drives the self-assembly via aromatic stacking interactions and that β-sheet fibrils are formed at sufficiently high concentration. Hydrogels were also observed at higher concentration, and these were used in preliminary cell culture experiments which showed viable bovine fibroblasts on Fmoc-RGD hydrogels but not Fmoc-GRD,^84^ consistent with the expected bioactivity of the integrin adhesion motif. In a similar vein, Fmoc-RGDS and scrambled Fmoc-GRDS have been investigated using a combination of experimental and modeling methods.^85^ As for the Fmoc conjugates to shorter tripeptide sequences, β-sheet fibrils and hydrogels were observed at sufficiently high concentration.^85^ The UV–vis and IR absorbance spectra of Fmoc-GRDS as well as Fmoc itself and of the fluorene building block, and the linear and circular dichroism spectra of these molecules along with Raman spectra, have been analyzed in detail and compared.^86^

A conjugate of Fmoc with a transthyretin core amyloid-forming peptide sequence YTIAALLSPYS forms β-sheet fibrils and time-resolved fluorescence spectroscopy showed excimer formation within the fluorophores in the fibrils.^87^

**Figure 4:**
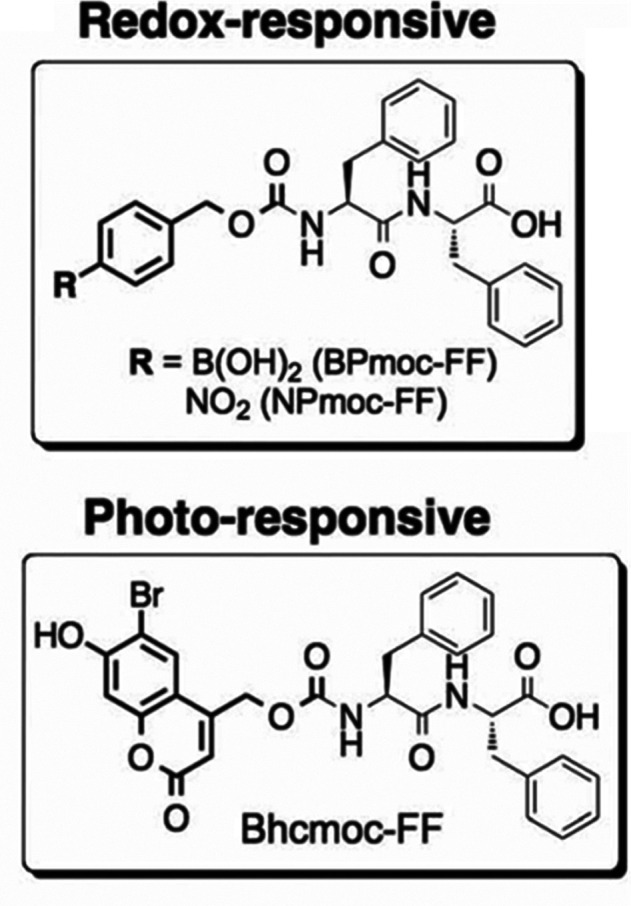
Stimulus-responsive FF derivatives. Reproduced with permission from ref (88). Copyright 2011 John Wiley and Sons.

A number of methoxycarbonyl derivatives have been linked N-terminally to FF to produce stimulus responsive hydrogels, specifically responsive to oxidation, reduction or light using *p*-borono-phenylmethoxycarbonyl (BPmoc), *p*-nitro-phenylmethoxycarbonyl (NPmoc), or 6-bromo-7-hydroxycoumarin-4-ylmethoxycarbonyl (Bhcmoc) (Figure 4).^88^ TEM on BPmoc-FF showed a fibrillar structure in the gels with a β-sheet structure based on XRD and Fourier transform infrared (FTIR) spectroscopy.

Fmoc tripeptides containing carnosine (β-alanine-histidine) at the C-terminus along with Fmoc-linked A, V, F, L, Y, I or M have been shown to form fibrillar hydrogels with a J-aggregate structure due to π-stacking of Fmoc groups.^89^ Fmoc peptides based on the IKVAV laminin sequence (laminin is a component of the extracellular matrix) that have been studied include Fmoc-IKVAV, Fmoc-DIKVAV and Fmoc-DDDIKVAV.^90^ These conjugates form fibrillar hydrogels under appropriate pH conditions, the nanofibrous matrix being of interest for proposed cell culture, since the gels have storage modulus values similar to those of soft tissues (being an order of magnitude higher for the D-containing conjugates).^90^

Fmoc-tetrapeptides Fmoc-XXFF where X indicates a polar residue, i.e., charged D, E, R or K or neutral N or Q have been prepared and studied.^91^ Fibrils were observed for all conjugates containing charged residues but not those with neutral N or F. Hydrogels were observed for all conjugates via pH switch using GdL. The cytotoxicity was also examined along with interactions with tethered lipid membranes.^91^ Fmoc-tetrapeptides DAAR or DGGR contain dialanine or diglycine substrates for enzymes including chymotrypsin, elastase or thermolysin. This has been used in an enzyme detection system based on covalent linking of the Fmoc peptides to PEGA (PEG/acrylamide copolymer) particles containing dextran which swell due to enzyme cleavage (which leads to D/R zwitterion formation) and dextran release.^92^

Fmoc-hexapeptides such as Fmoc-ILVAGK, Fmoc-LIVAGK, and Fmoc-AIVAGK containing cationic residues are able to form hydrogels, whereas hydrogels are not observed for conjugates with FF added in the sequence in Fmoc-FFILVAGK etc.^93^ This highlights the importance of the balance between aromatic interactions and others such as electrostatic forces. The conformation and critical aggregation concentration of these conjugates was carefully determined.^93^ Mixing these Fmoc-peptides with Fmoc-FF leads to self-sorted fibrillar hydrogels with tunable, wide range of modulus.^94^ The hydrogels have generally excellent cytocompatibility, pointing to their potential use as synthetic extracellular matrices.

Fmoc-depsipeptides containing K or D side chain sequences have been shown to form fibrils and gels, the gelation time reducing significantly in the presence of NaCl and/or PBS compared to water.^95^

### 2.4 Enzyme Treatment and Hydrogelation of Fmoc-Peptides

Enzyme-instructed self-assembly (EISA),^10^ discussed in detail in section 3.2, has been used to trigger hydrogelation by alkaline phosphatase (ALP) treatment of Fmoc-pY or Fmoc-pY with an Fmoc/lysine derivative.^96,97^ Gelation is prevented by phosphatase inhibitors, inspection of the sample providing a simple visual assay for the enzyme inhibitor.^96^ A model for the packing of hydrophobic Fmoc-Y-OMe molecules in the supramolecular fibrils formed after phosphatase treatment, driven by Fmoc stacking was proposed.^98^ Later, the same method was used with Fmoc-FFpY (pY: phosphotyrosine) to give Fmoc-FFY, as part of a study with mixtures of chemiluminescent molecules (also prepared by ALP treatment of precursors).^99^ Fmoc-FFY and Fmoc-FFGGGY were used in hydrogels which were photo-cross-linked to provide greatly improved stiffness (10^4^-fold increase in storage modulus).^100^ Two tyrosine residues in close proximity can be photo-cross-linked using Ru(bpy)_3_^2+^ (bpy: bipyridine) catalysis, and it was established that the β-sheet fibrillar structure was retained during cross-linking.

The packing within fibrillar hydrogels such as Fmoc-YL can be tuned by thermal annealing, which influences the balance between hydrogen bonding and π-stacking interactions, as revealed by detailed FTIR, NMR, and CD measurements.^101^ The influence of the chemical nature of the linker between the fluorenyl group and a dipeptide has been examined using four fluorenyl derivatives of different flexibility and YL as model dipeptide.^102^ It was demonstrated that the methoxycarbonyl group present in Fmoc is optimal, being a rigid linker of sufficient length between the bulky aromatic and peptide groups. Fmoc-YL was also used as a model system for examining the influence of salts from the Hofmeister series on the conformation and self-assembly of hydrogel-forming Fmoc-peptides (different morphologies were observed with different anions), as well as the gel properties including melting temperature and modulus.^103^ Anions were also found to promote hydrophobic interactions, and thus self-assembly, in Fmoc-YL, Fmoc-VL, and Fmoc-LL, although this occurs to a lesser extent with Fmoc-AA which is less hydrophobic. The effects of Hofmeister series salts on the subtilisin-induced self-assembly (vide infra) of Fmoc-YL-OH were also elucidated, and distinct modes of chiral organization and self-assembly were revealed.^104^ Salt effects on the enzyme network were also uncovered, this in turn affecting the kinetics of enzyme activity and the resultant nucleation and growth of nanostructures. It was also shown that the enzyme-catalyzed assembly processes are kinetically controlled, and thermodynamically favored states can be accessed by heating/cooling treatment.^104^

**Figure 5:**
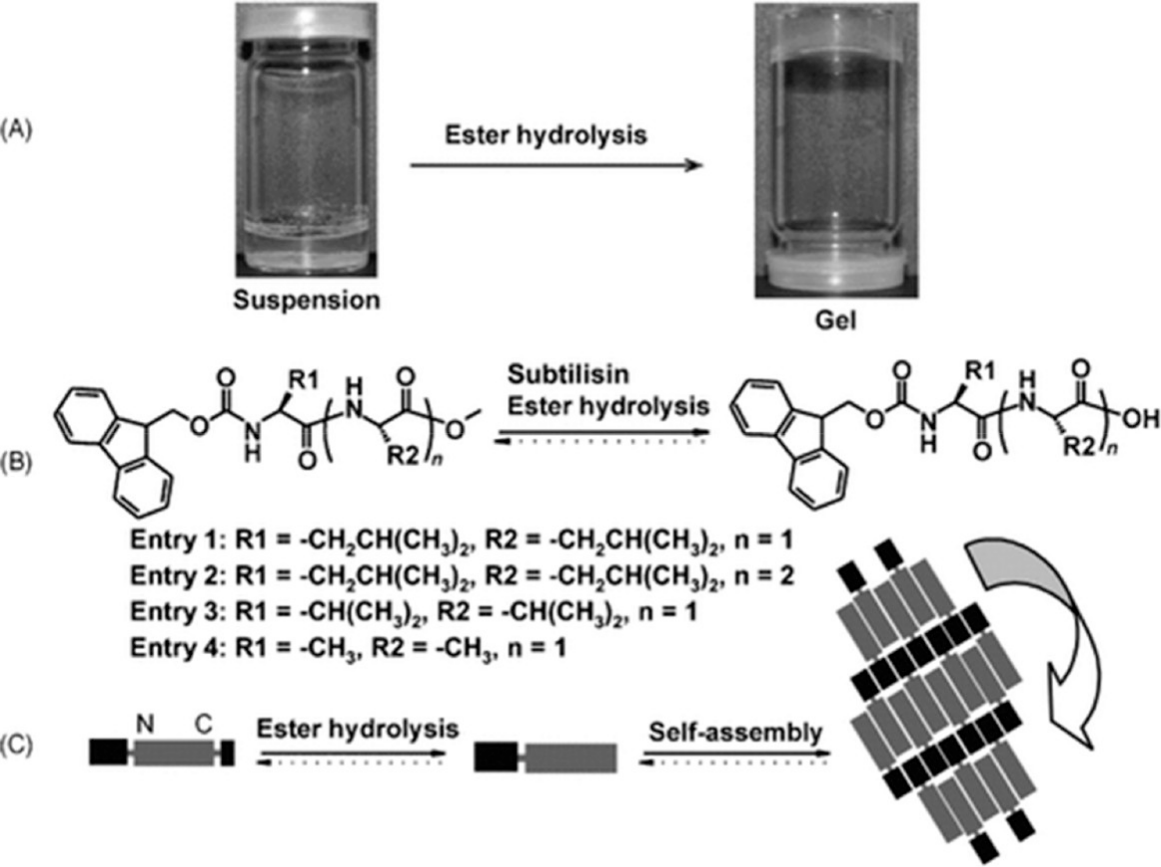
Hydrolysis of Fmoc-dipeptides using subtilisin. (A) Sol–gel transition for Fmoc-LL. (B) Reaction scheme showing structures of conjugates studied. (C) Schematic showing self-assembly process. Reproduced with permission from ref (105). Copyright 2008 John Wiley and Sons.

Enzymatic hydrolysis can be used to remove C-terminal esters to drive self-assembly of Fmoc-peptides. This has been extensively explored by the Ulijn group.^105,106^Figure 5 shows the hydrolysis process along with images of a hydrogel formed by Fmoc-LL after treatment with subtilisin and a schematic of the self-assembly process.^105^ The hydrogels comprise fibrils or nanotubes depending on the peptide. The effect of enzyme concentration in improving supramolecular ordering was probed for a series of Fmoc-dipeptides including Fmoc-FF, Fmoc-YL, and others.^106^ It was shown that increase of enzyme concentration leads to bundling of the peptide conjugates and greater fibril network connectivity and rigidity, with reduced mobility of enzyme clusters which reduces discontinuities in fibril assembly. Hydrolysis of Fmoc-L_3_-OMe using subtilisin gives rise to Fmoc-L_3_-OH nanotubes, xerogels of which show significant electrical conductivity, due to π-stacking interactions which were examined by XRD and MD simulations.^107^

**Figure 6:**
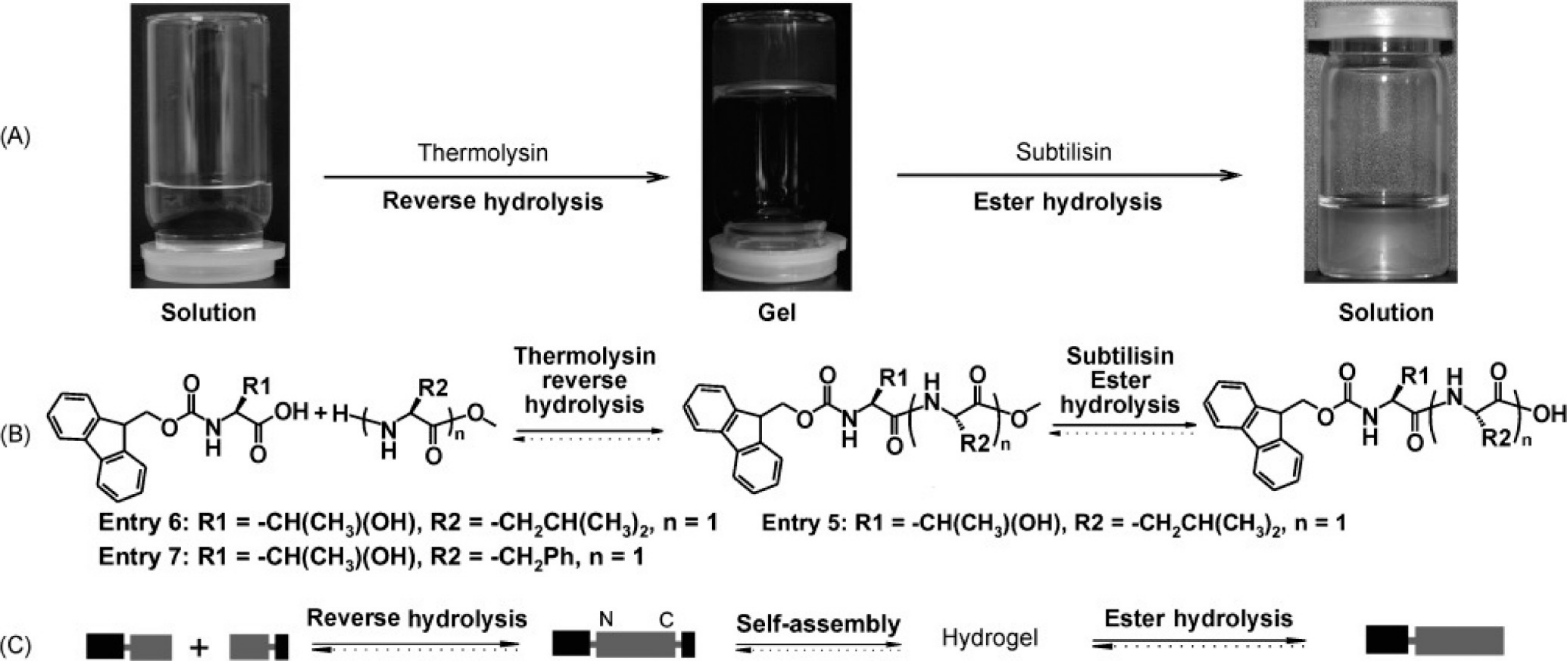
Reverse hydrolysis of Fmoc peptides with free amino acid (peptide) methyl esters, followed by ester hydrolysis. (A) Illustration of sol–gel–sol transitions for Fmoc-T-OH and l-OMe. (B) Reaction schemes for peptides and amino acids. (C) Schematic showing self-assembly process. Reproduced with permission from ref (105). Copyright 2008 John Wiley and Sons.

Reverse hydrolysis can also be used to condense Fmoc-peptides with amino acids or peptides, leading to gelators as shown in Figure 6, which shows a sol–gel–sol process for Fmoc-T-OH and l-OMe which undergo condensation through thermolysin-driven reverse hydrolysis.^105^ The resulting Fmoc-peptide-ester forms a fibrillar hydrogel and can be hydrolyzed with subtilisin, recovering a solution state.

**Figure 7:**
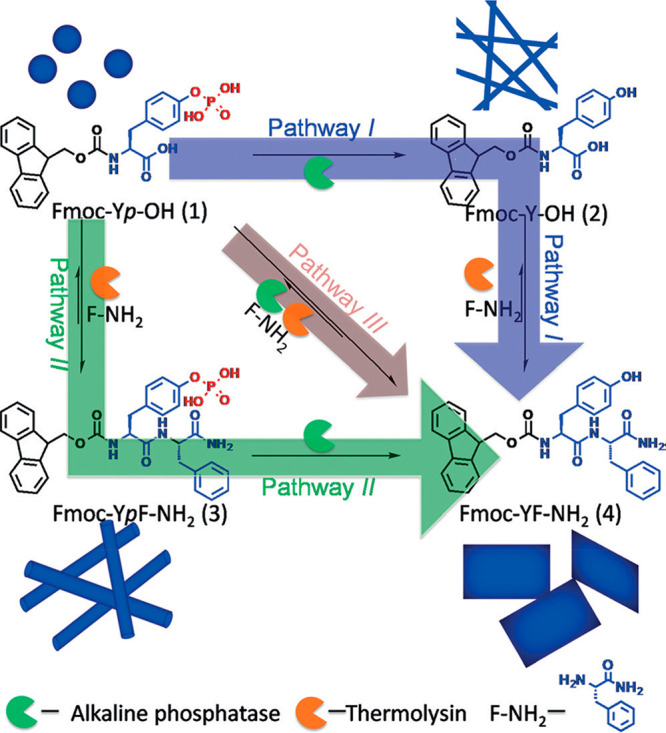
Enzymatic cascades with Fmoc-pY-OH and F-NH_2_ to produce Fmoc-YF-NH_2_ via three pathways as shown, using the two enzymes shown. Reproduced with permission from ref (109). Copyright 2017 John Wiley and Sons.

The hydrolysis/reverse hydrolysis equilibrium can exist under thermodynamic control, as shown by experiments on mixtures such as Fmoc-F with F_2_ in the presence of thermolysin.^108^ A distribution of Fmoc-peptides is formed which was analyzed using HPLC, with Fmoc-F_3_ predominant in this case, over the time scale examined. Similar results were obtained with other Fmoc-amino acids and nucleophiles. Long-time remodeling of the product distribution (dynamic combinatorial library) was observed with Fmoc-L/L_2_ due to continued (reverse) hydrolysis. It was also proposed that nucleation of fibrils occurs locally from regions enriched in enzyme.^108^ The Ulijn group has also demonstrated enzymatic cascades using Fmoc-peptides as shown in the schematic in Figure 7.^109^ Starting from Fmoc-pY-OH, it was possible to obtain Fmoc-YF-NH_2_ via three pathways using alkaline phosphatase (to dephosphorylate) and/or thermolysin (to promote condensation with F-NH_2_) in different orders. The morphologies observed by TEM are sketched in Figure 7 and the final state of the product (gel or suspension) is dependent on the pathway. Dipeptide F_2_ also undergoes enzyme-induced self-assembly with a range of Fmoc-amino acids, as shown by a study on gel formation after thermolysin treatment.^110^

Condensation of Fmoc-amino acids (Fmoc-CA or Fmoc-K, CA: cysteic acid) with F-NH_2_ using thermolysin has been performed in the presence of differently charged polysaccharides.^111^ This leads to a dynamic peptide library comprising Fmoc-CAF or Fmoc-KF. In the presence of cationic polysaccharide chitosan, amplification of nanosheet-forming Fmoc-CAF is noted, whereas in anionic heparin, Fmoc-KF that bears the oppositely charged K residue and that forms nanotubes is produced.^111^ Coassembly of proteins β-lactoglobulin or bovine serum albumin with Fmoc-YN, Fmoc-YS, Fmoc-YL, or Fmoc-VL was probed using light scattering and other methods, which revealed the formation of fractal-like clusters of proteins coassembling with the Fmoc-dipeptide fibrils.^112^ The proteins influence the chiral organization of the peptide conjugates and can produce enhanced ordering, and the mechanical properties of nanostructured hybrid gels can also be tuned depending on the mixture composition.

Thermolysin-induced condensation of Fmoc-SF, Fmoc-SL, Fmoc-TF, or Fmoc TL with F-OMe or l-OMe occurs under thermodynamic control, giving rise to extended aggregate structures based on π-stacking of Fmoc, with subtle differences in molecular organization.^113^ For Fmoc-S undergoing thermolysin-catalyzed condensation with F-OMe, nanosheets were reported as the thermodynamic product as opposed to the more usual fibrillar structures.^114^ The nanosheets with β-sheet packing were proposed to form with sequestered hydrophobic interior and exterior decorated with serine hydroxyl groups.

With the aim to create simplified mimetics of natural enzymes, short peptides capable of catalyzing reactions that produce Fmoc-dipeptides (via a process similar to that catalyzed by thermolysin) were screened via phage display of 10^9^ dodecapeptides, analyzed for catalytic activity in condensing Fmoc-T with l-OMe.^115^ Successful catalysis leads to aggregation of product, and the aggregates could then be separated by centrifugation.

## 3 Naphthalene

### 3.1 Naphthalene–Dipeptide Conjugates

**Figure 8:**
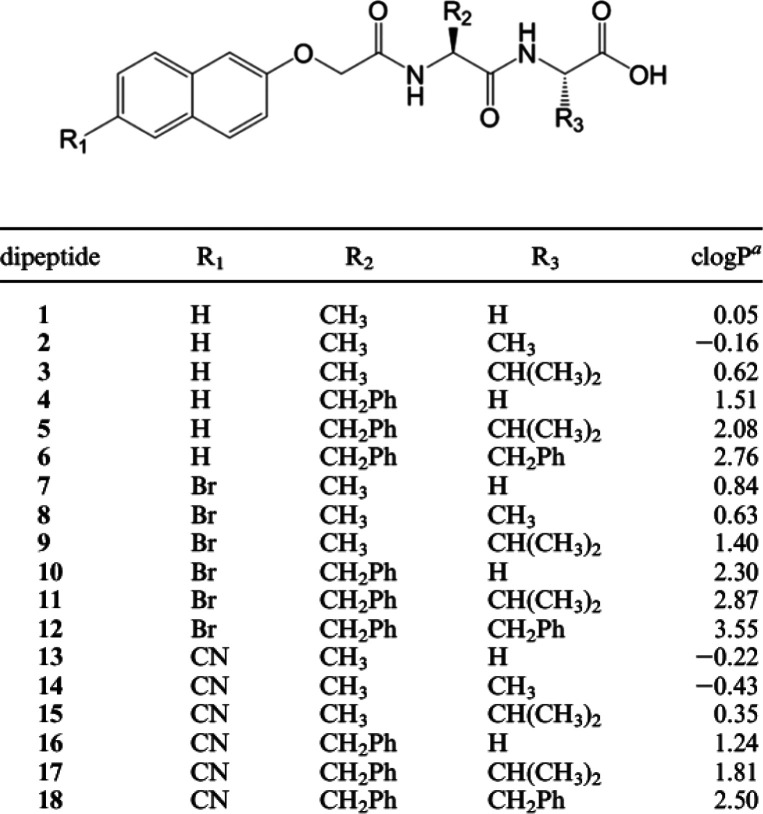
Naphthalene dipeptide derivatives studied by Chen et al. Reproduced from ref (117). Copyright 2010 American Chemical Society.

The hydrogelation ability of a series of naphthalene-based dipeptide conjugates (dipeptides linked to N-terminal naphthalene via a (2-yloxy)acetic acid unit or other linker) has been examined.^116^ Hydrogels were observed for Nap-GG, Nap-Ga, Nap-GA, Nap-GS, at low pH, although a gel–sol transition is observed on heating, at around 50 °C. The gels comprise fibrillar structures and show good cytocompatibility with HeLa cells.^116^ A considerable number of naphthalene–dipeptides (Figure 8) have been screened for their ability to form a hydrogel, and adsorption isotherm measurements were performed, which provided values for cmc (critical micelle concentration), air–water partition coefficient, and area per molecule for the compounds.^117^

Nap-FF is a hydrogelator as are a range of other Nap-FF-based derivatives.^118^ As for Fmoc conjugates discussed in the previous Section, hydrogels can be prepared by different methods from a solution at an initial pH including via addition of salt, by pH reduction, upon addition of acid or hydration of an initial solution in organic solvent.^119^ This leads to differences in the fibril morphology (as detected by microscopy and small-angle scattering) and the gel mechanical properties.^119^ It is also possible to create hydrogels using a photoacid generator in the presence of UV light, as demonstrated for Nap-FF and other Nap-dipeptide derivatives and a Fmoc-dipeptide.^120^ The photoacid generator is used to lower the pH of a peptide conjugate below the apparent p*K*_a_ of the gelator, leading to gelation.

Addition of divalent cations at high pH has been used to produce hydrogels of Nap-FF, with a high value of the storage modulus.^121^ This was ascribed to the formation of salt bridges between the carboxylates on the wormlike micelles that are formed at high pH in this system. The concentration-driven transition from spherical to worm-like micelles in Nap-FF solutions has been studied, along with the formation of gels formed by addition to Ca^2+^ to a solution of wormlike micelles.^122^ Nap-FF forms a transient gel at low pH (pH < 5.7), then addition of NaOH drives a gel–sol transition, and an out-of-equilibrium state can be produced by using the urease-catalyzed hydrolysis of urea (section 2.1) to increase pH.^123^ Homogeneous gelation can then be driven at high pH by the addition of Ca^2+^ ions. It is possible to produce a system that shows dynamic degelation, then regelation by adjustment of the concentrations of Nap-FF, urease, urea, and Ca^2+^.^123^ The mesh size of Ca^2+^-induced Nap-FF gels has been estimated using pulsed-field gradient NMR via measurements of the self-diffusion of dextrans with different molar masses and hence radii of gyration (covering the range of expected pore sizes within the fibrillar gel network).^124^ Heat–cool treatment of high pH solutions of Nap-FF can also be used to produce viscous solutions of wormlike micelles (hollow cylinders), the mechanical properties of which have been examined during capillary breakup under extensional deformation.^125^ Addition of CaCl_2_ after the heating step gives a more transparent gel. An analogue of Nap-FF, containing 1,2,3,4-tetrahydronaphthalene forms gels in the presence of Ca^2+^ that can be drawn or spun into “noodles” (macroscopic fibers), the mechanical properties of which were analyzed.^126^ The effect of different alkaline hydroxides on the solution morphology and viscosity of Nap-FF has been examined.^127^

The assembly process of BrNap-AV has been investigated with solution state NMR spectroscopy.^128^ Using molecular probes (^14^NH_4_^+^ and isopropanol-*d*_8_) in residual quadrupolar coupling (RQC) measurements, as well as the NMR relaxation rates of ^23^Na^+^, it was possible to obtain information on the charge, hydrophobicity, and molecular structure within aggregates which are silent in conventional ^1^H NMR spectroscopy due to low mobility of the molecules. A multistage process driven by neutralization as pH is reduced was uncovered.^128^ As well as these RQC and saturation transfer difference probe molecule methods (using a range of positive ion, hydrophobic, ion binding, and NMR pH indicator probes), chemical shift imaging has also been used to study the binding of Ca^2+^ to Nap-FF, BrNap-AV, and Fmoc-LG, following the formation of hydrogels along chemical gradients.^129^ Self-sorting is observed for gels of BrNap-AV with Nap-FF due to the distinct p*K*_a_ values for the two molecules which facilitates separate self-assembly induced by the GdL hydrolysis method of pH reduction, the former conjugate aggregating first at a higher pH.^130^

The structure of Nap-FF fibrils has been investigated in detail using small-angle scattering and MD simulations.^131^ In particular, contrast variation SANS using a range of selectively deuterated Nap-FF compounds was employed to gain insight into the molecular organization within the fibrils, which it is proposed have a hollow cylinder structure in the solution state, although this nanostructure is lost as gelation occurs.^131^ Caution is required since, in certain cases, isotope effects on self-assembled structures are observed for peptide conjugates when comparing small-angle scattering data and/or cryo-TEM for H_2_O and D_2_O solutions, i.e., the same structure is not always observed in the two solutions.^132^ SANS has also been used to probe the effect of drying on the fibril network of Nap-FF and related conjugates, via changes in H or D content, following removal of H_2_O or D_2_O from gels of partially or undeuterated Nap-FF (or 6Br-Nap-FF) to produce xerogels.^133^ This showed that drying does affect the fibril network. Addition of coumarin to a gel of a Nap-FF analogue [containing 3,8a-dihydro-2*H*-chromen-2-one] leads to coassembly, and exposure to UV leads to coumarin dimerization which affects the molecular packing in the fibrils and the stiffness (shear modulus).^134^ Drop-casting of Nap-FF (or BrNap-FF or BrNap-AV) in a high pH solution onto a low pH subphase leads to films comprising a fibril network.^135^

**Figure 9:**
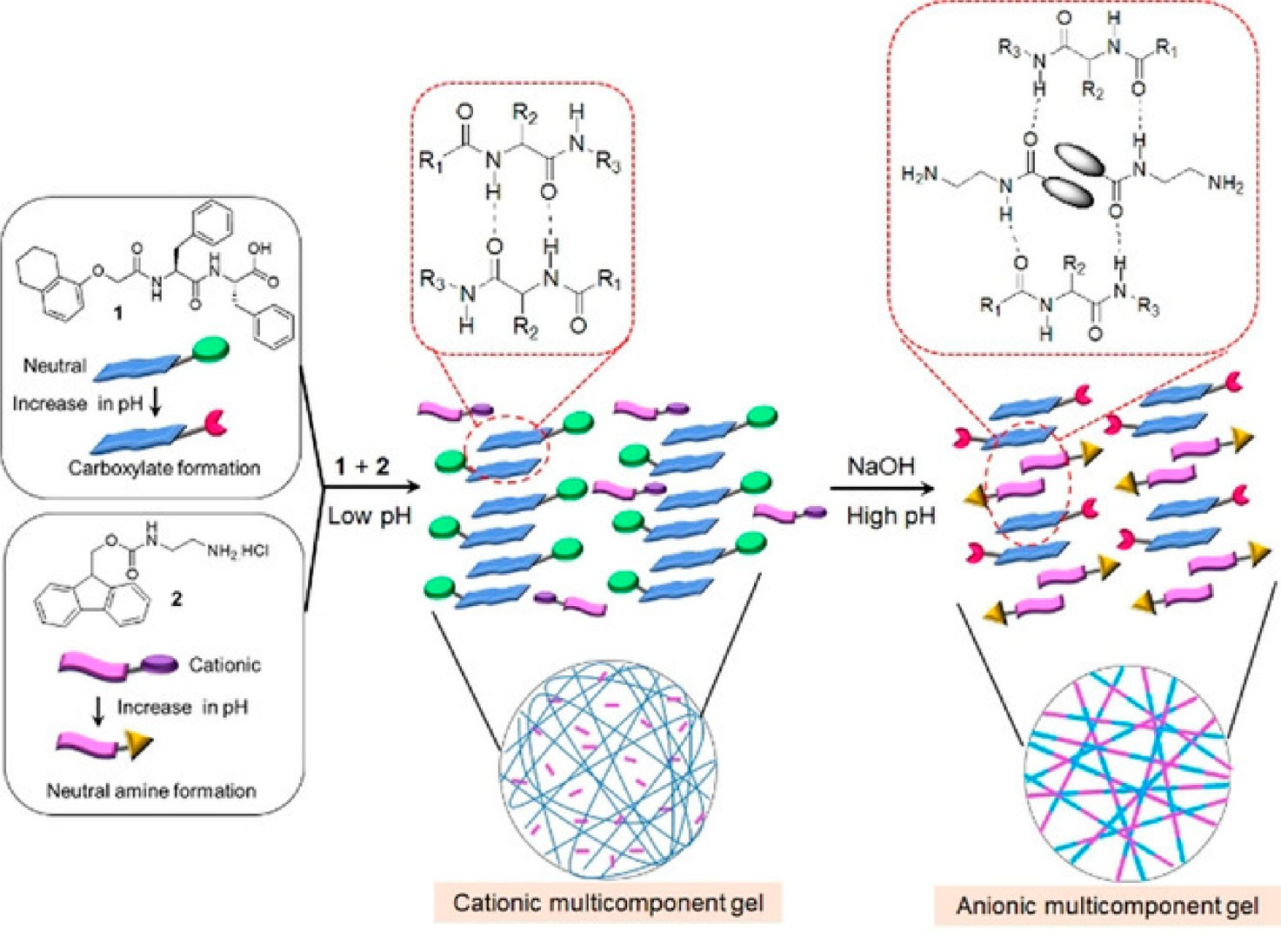
Coassembly of Nap-FF with Fmoc-amine leads to differences in molecular packing in gels at different pH values.^136^

Coassembly of Nap-FF and a Fmoc-amine (Figure 9) leads to two-proposed modes of aggregation in a multicomponent gel, depending on pH.^136^ Nap-FF-OH coassembles with a pyrene ammonium salt to form a fibrillar gel.^137^ The gel can be cross-linked using hydrolysis of carbodiimide EDC [1-ethyl-3-(3-(dimethylamino)propyl)carbodiimide], which leads to enhancement of the gel shear moduli.

**Figure 10:**
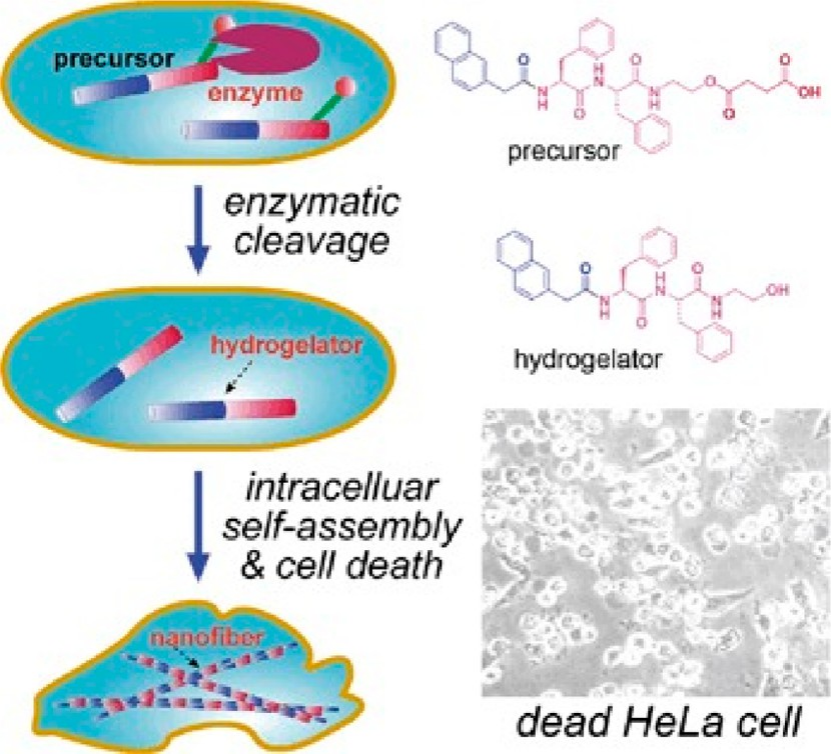
Aggregation of Nap-FF based derivative shown into fibrils, in response to endogenous esterases in HeLa cells, leading to significant cytotoxicity. Reproduced with permission from ref (138). Copyright 2007 John Wiley and Sons.

Nap-FF has been used as a motif in a range of conjugates developed by the group of Bing Xu with novel bioactivities. In an early example, Nap-FF was linked to a C-terminal butyric acid unit, which is a substrate for esterase.^138^ Intracellular esterase activity leads to self-assembly of Nap-FF-OH into fibrils which were found to be selectively cytotoxic to HeLa cells, but not NIH3T3 fibroblasts (Figure 10). Nap-F with a related C-terminal extension also forms hydrogels after exposure to esterase.^139^ The hydrogel is anisotropic, as shown by the presence of birefringence when viewed between crossed polars. Nap-FF forms fibrils in aqueous solution and its crystal structure has been reported, enabling a model for the molecular packing in the fibrils to be proposed.^140^ This conjugate shows selective cytotoxicity to glioblastoma cells due to its propensity to aggregate into fibrils, as opposed to the lack of toxicity to neuronal cells.^140^ The selective inhibition of glioblastoma cells was ascribed to interaction with microtubules, as revealed by confocal cell imaging using tubulin tracker dye. Nap has also been conjugated to the C-terminus of diphenylalanine, with 3 different length linkers and the resulting conjugates are also fibril-forming hydrogelators, with selective activity against cancer cells (HeLa compared to noncancerous Ect1/E6E7 cells).^141^

Nap-FF has also been used as a self-assembly scaffold to attach cationic lysine or ornithine residues in the development of antimicrobial nanomaterials.^142^ The conjugates form fibrillar hydrogels and the greatest antimicrobial activity was displayed by Nap-FFKK against *Staphylococcus epidermis* biofilm. However, this compound is unfortunately also cytotoxic to murine fibroblasts and also exhibited substantial hemolysis against equine red blood cells.^142^ Cytotoxicity against mammalian cells obviously needs to be minimized along with substantial antibacterial activity, for a practically useful antimicrobial agent. Antimicrobial activity is also observed for Nap-FF mixed with dopamine.^143^ The latter undergoes autoxidation in the presence of molecular oxygen to form quinones, with H_2_O_2_ as a byproduct, which has antimicrobial properties. Antimicrobial activity of gels incorporating dopamine was observed at high pH for *Staphylococcus* species.^143^

A range of conjugates comprising naphthalene derivatives (with Br-, CN-, or H at the 6-position) linked to dipeptides AV has been prepared to examine gelation propensity, after preparation using pH reduction by GdL hydrolysis.^144,145^ The nature of the naphthalene derivative influences hydrogen bonding and π-stacking and hence molecular packing, while the chirality of the assemblies probed by CD depends on the dipeptide sequence. Nap-AA forms gels (by GdL treatment of an initial pH 10.5 solution) in which crystals form slowly, and the kinetics of this process were analyzed by time-resolved small-angle X-ray scattering/wide-angle X-ray scattering.^146^ The ability of Nap-AA to form crystals from a hydrogel had previously been noted in a screening study on Nap-dipeptide systems comparing gel vs crystal formation.^147^ The structure in the crystal is the same as that in the dried gel, although it differs from that of hydrated fibrils.

Naphthalene-alanine derivatives with different C-termini form fibrillar structures and the resultant hydrogels are injectable under appropriate conditions.^149^ The hydrogels can be used to entrap doxorubicin (Dox) and show sustained slow release of this anticancer drug and improved antitumor activity was observed for the Dox-loaded gel using a mouse model.

Longer conjugates of naphthalene/pY-containing peptides include those based on peptides from insulin growth factor.^150^ Self-assembly and conformation were observed to be temperature-dependent.

### 3.2 Naphthalene–Peptide Conjugates: Enzyme Treatment

Enzyme-instructed self-assembly (EISA) has emerged as a powerful tool to create responsive bionanomaterials that has been developed by the groups of Xu, as reviewed elsewhere.^10,151,152^ This method has particularly been applied to generate naphthalene–peptide fibrillar hydrogelators by dephosphorylation of pY-containing peptides. Nap-GFFpY-OMe forms hydrogels in the presence of phosphatase and coassembly with ovalbumin leads to gels incorporating this immune stimulating vaccine adjuvant. A fibrillar structure is observed for the gels.^153^ The hydrogel improves antigen uptake and leads to dendritic cell maturation, and accumulation of antigen in lymph nodes, stimulating germinal center formation.^153^ A hydrogel based on the d-peptide analogue, and incorporating ovalbumin, was found to inhibit tumor growth to a greater extent than its l-peptide analogue.^153^ The l-peptide Nap-GFFpY-Nme (with C-terminal methyl amide group) also forms fibrillar hydrogels after phosphatase treatment, and coassembly with HIV DNA yields a gel able to raise both humoral and cellular immune response against HIV, thus having potential in the development of HIV vaccines.^154^ As mentioned above, conjugates of Nap with d-amino acid residues have enhanced stability against proteolysis. Nap-Gfffy has been shown to form supramolecular hydrogels which can incorporate antigens (through the thioxotropic property of the gels) such as ovalbumin and which can thus act as vaccine adjuvants.^155^ This was shown via antibody titers, and quantification of immunoglobulin and cytokine production showing cytotoxic T-cell stimulation. Inhibition of tumor growth in vivo (mouse model) was observed.^155^

The d-amino acid Nap-Gff(py) (py: d-phosphotyrosine) forms hydrogels in the presence of Ca^2+^ ions as well as through EISA.^156^ This gel protects phosphorylated antigens from dephosphorylation, thus increasing the proportion of antibodies to phosphorylated proteins.

**Figure 11:**
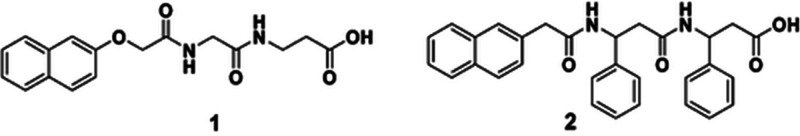
Naphthalene conjugates containing dipeptides with one or two β-amino acids. Reproduced with permission from ref (158). Copyright 2006 Royal Society of Chemistry.

Naphthalene conjugates containing non-natural variants of FF such as dipeptides of d-phenylalanine and s-β^3^-H-phenylglycine (s-β^3^-HPhg), and l-4-fluorophenylalanine (l-fPhe) also form fibrillar hydrogels that are resistant to proteolysis by proteinase K and controlled release of radiolabeled iodine salts and ligands was observed in vivo.^157^ Other Nap derivatives containing β-amino acids were designed to improve resistance to proteolysis, including the compounds shown in Figure 11 containing alanine and β^3^-alanine (**1**) or two β^3^-phenylalanine (**2**) residues.^158^ These form fibrillar hydrogels. Nap-s-β^3^-HPhg-s-β^3^-HPhg-pY-OH forms a hydrogelator after treatment with phosphatase, as does Nap-FFpY-OH.^159^ Gelation is observed in aqueous solutions and in blood (for the β-peptide derivative), and low in vivo toxicity of the gels was reported.

Nap-FFKY has been linked via the lysine ε-amino group to an enterokinase substrate sequence DDDDK, related to the FLAG tag, an affinity tag for protein purification.^160^ The conjugation leads to a T-shaped conjugate which forms a fibrillar hydrogel after enterokinase cleavage. A similar conjugate bearing a fluorescent NBD [4-nitro-2,1,3-benzoxadiazole] group was also developed for cell labeling, which combined with cell compartment dyes, enabled imaging of the localization of ENTK-cleaved fibrils (the conjugate forms micelles prior to enzyme cleavage) on mitochondria in HeLa cells (but not cancer cells).^161^ Delivery of protein or the anticancer drug doxorubicin cargo via branched peptide micelles was also demonstrated.^161^ The d-amino acid conjugate Nap-ffk(py) has been used as a scaffold to attach NBD or the anticancer drug taxol (both via ε-amino lysine).^162^ Enzymatic dephosphorylation leads to fibril formation and hydrogelation. Self-assemblies of the NBD derivative localize around the endoplasmic reticulum, as revealed by cell imaging by confocal microscopy.^162^

**Figure 12:**
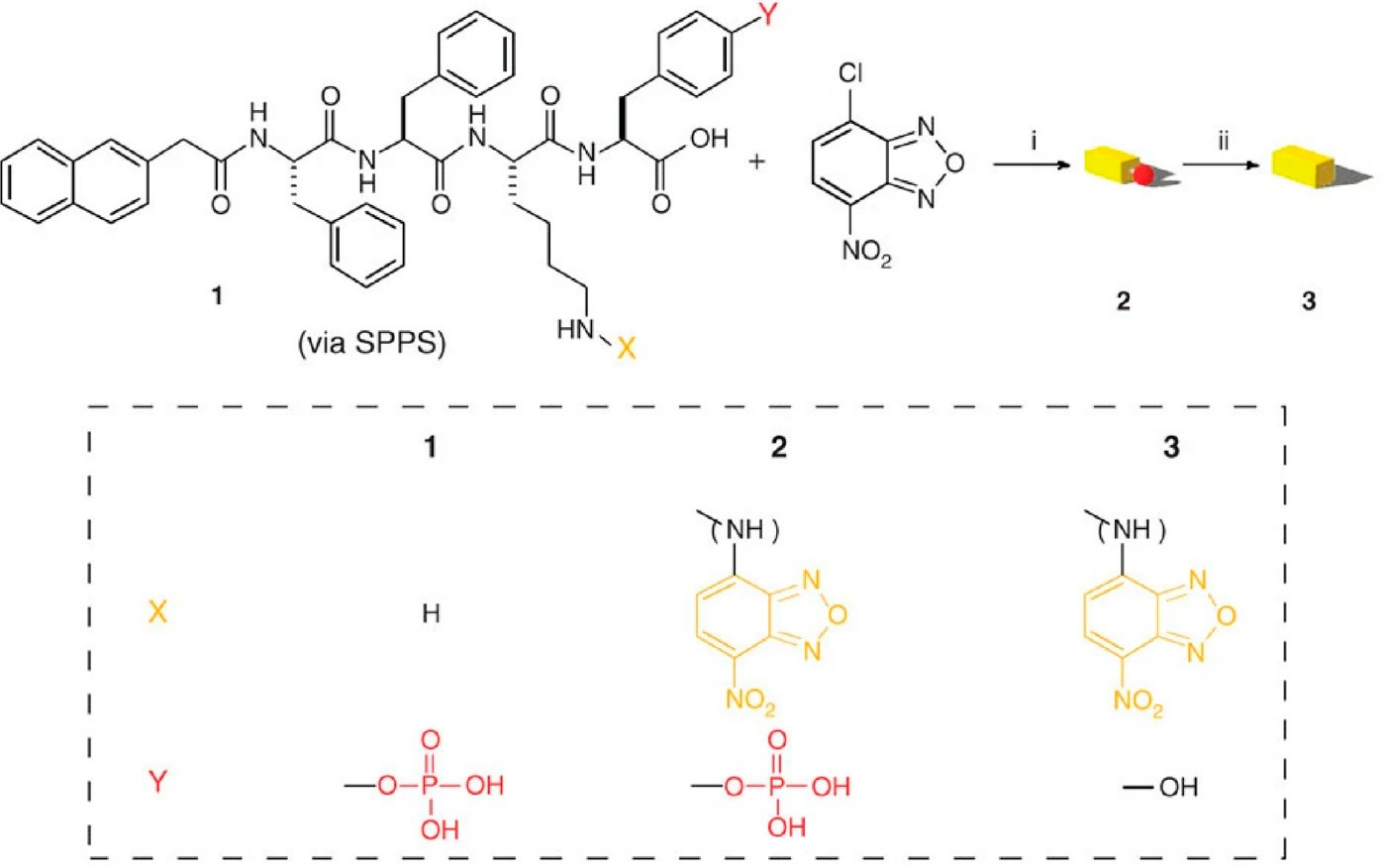
Fluorescently labeled naphthalene–dipeptide derivative that undergoes EISA in living cells. (i) Na_2_CO_3_, methanol, water, 50 °C, 2 h; (ii) alkaline phosphatase. Reproduced with permission from ref (163). Copyright 2012 Springer Nature.

A naphthalene-FF derivative with NBD linked via a lysine side chain and containing phosphotyrosine (Figure 12) undergoes EISA in living cells.^163^ Dephosphorylation occurs in vivo to produce fibrillar hydrogels within the HeLa cells studied and thus fluorescently labeled cells.

**Figure 13:**
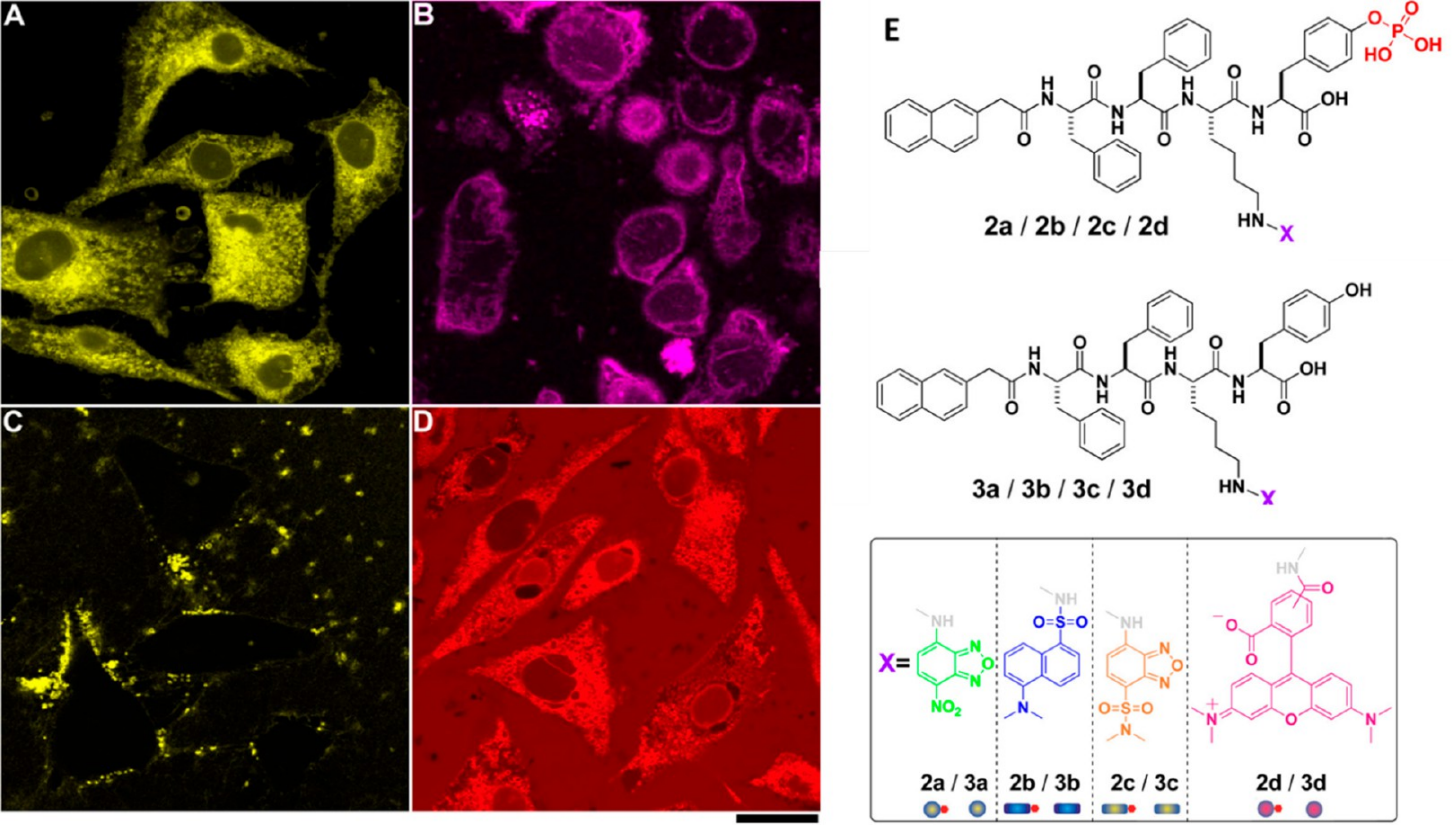
Fluorescent confocal microscope images of HeLa cells incubated in PBS buffer with Nap derivatives with fluorophores: (A) NBD, (B) dansyl, (C) 4-(*N*,*N*-dimethylsulfamoyl)-2,1,3-benzoxadiazole (DBD), and (D) rhodamine. Scale bar = 25 μm. (E) Molecular structures **2a**, **3a** NBD, **2b**, **3b** DNS, **2c**, **3c** DBD, **2d**, **3d** rhodamine. Reproduced from ref (164). Copyright 2013 American Chemical Society.

The work was developed by attaching differently colored fluorescent dyes to Nap-FFKpY (via the lysine ε-amine group), leading to distinct cell labeling, shown for example in Figure 13.^164^ The fluorescent imaging reveals that self-assembly induced by the activity of cellular phosphatases influences the localization of these conjugates in the cellular environment. In addition, cell viability tests suggest that the states and the locations of the molecular assemblies control the phenotypes of the cells.

**Figure 14:**
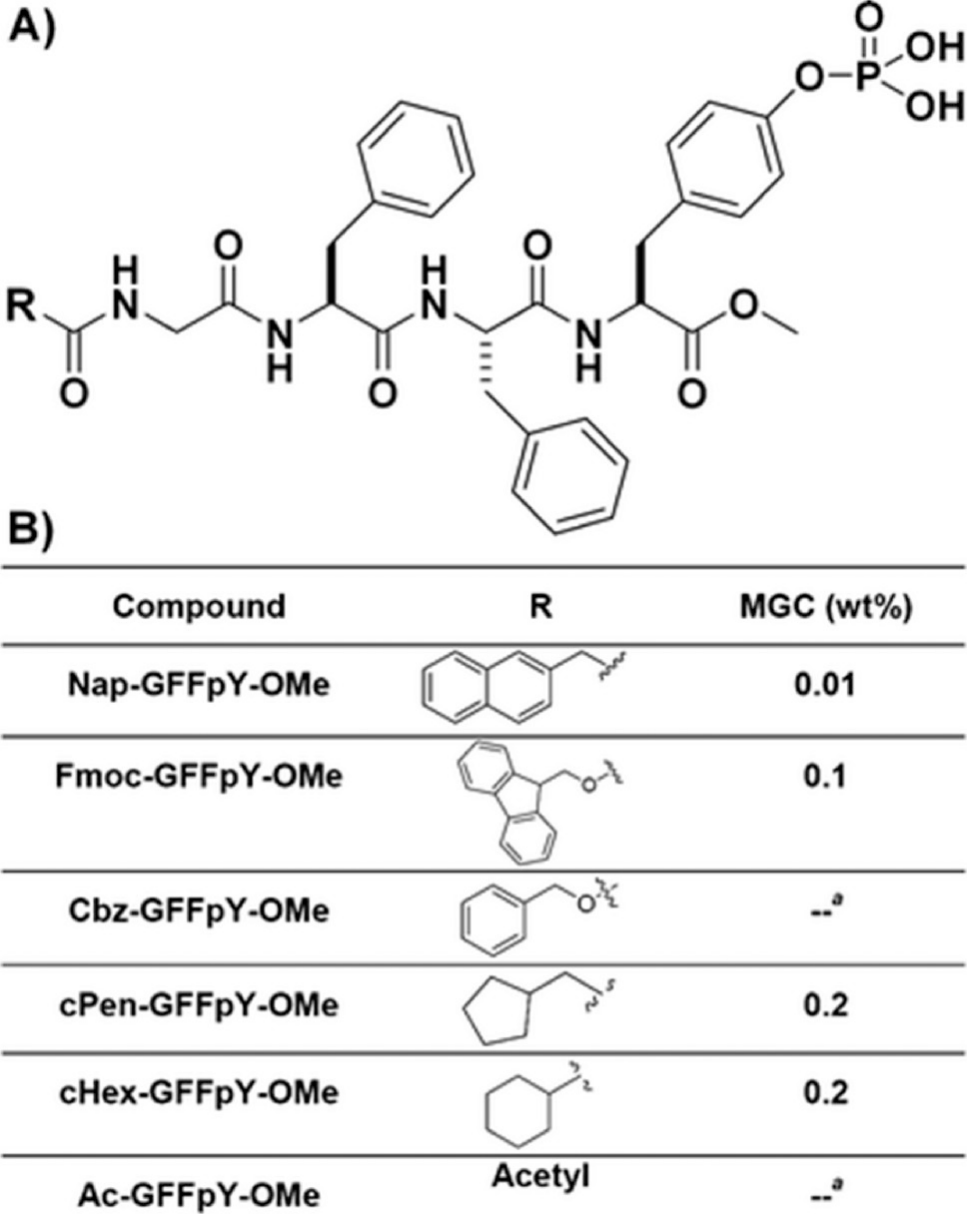
Conjugates with N-terminal aromatic groups shown and peptide GFFpY-OMe: (A) molecular structures; (b) N-terminal groups and minimum gelation concentration (MGC) values. Reproduced with permission from ref (148). Copyright 2011 Royal Society of Chemistry.

The conjugate Nap-GFFY (containing a model peptide sequence) can form hydrogels based on β-sheet fibrils, the formation of which is driven by aromatic stacking of the naphthalene unit as well as the aromatic residues.^165,166^ A modified version of this molecule, Nap-GFFY-OMe forms a hydrogel at very low concentration (0.01 wt %) after EISA from phosphatase treatment of Nap-GFFpY-OMe.^148,167^ The minimum gel concentration was compared to a number of related naphthalene-terminated short peptide derivatives bearing pY or a series of compounds with AG-GFFpY-OMe (AG = aromatic group; see Figure 14) after dephosphorylation.^148^ Nap-GFFY was used to produce hybrid hydrogels with high storage modulus based on conversion of Y into DOPA (3,4-dihydroxyphenylalanine) using mushroom tyrosinase which acts as a linker of peptide fibrils, and also containing silica nanoparticles.^168^ Nap-GFFY is a fibril-forming hydrogelator.^166^ The gel properties were compared to those of Fmoc-GFFY, PTZ-GFFY (PTZ: phenothiazine), and Cbz-GFFY (Cbz = benzyloxycarbonyl), with PTZ-GFFY forming hydrogels at a very low concentration.^166^ Hydrogelation was also examined for Nap-GFFYGGXO where O denotes hydroxyproline and X is K, E, S, A, or P, GXO being a collagen repeat sequence.^165^ Several of the hydrogels were able to support NIH 3T3 cell culture, the best with X = A having properties similar to collagen in this regard.

EISA can be modulated by ligand–receptor interactions as shown in an investigation of the behavior of Nap-FFpYGGaa which contains pY, along with di-d-alanine (aa) as receptor for the ligand vancomycin.^174^ ALP activity converts the precursor into Nap-FFYGGaa which forms fibrils, however in the presence of the ligand, aggregates comprising precipitates of shorter fibrils are formed. Vancomycin is unable to interact with the fibrils within hydrogels formed in the former case, but the hydrogels can be broken up using surfactants, which facilitates ligand–receptor interactions.^174^

**Figure 15:**
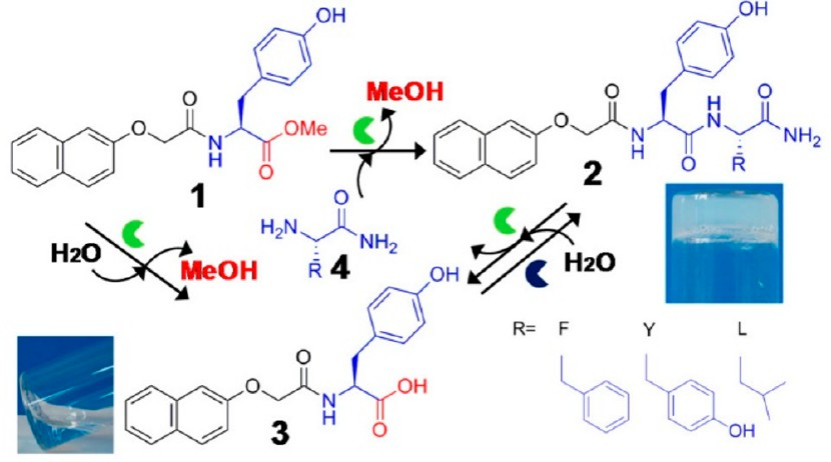
Competitive acylation and amide hydrolysis reactions in Fmoc-Y-OMe (**1**) + X-NH_2_ (**4**, X = F, Y or L) system. The green symbol represents α-chymotrypsin and the dark blue one thermolysin. Product **2** is a temporary hydrogelator (gel image shown) which is hydrolyzed to **3** and **4**. Reproduced from ref (175). Copyright 2013 American Chemical Society.

**Figure 16:**
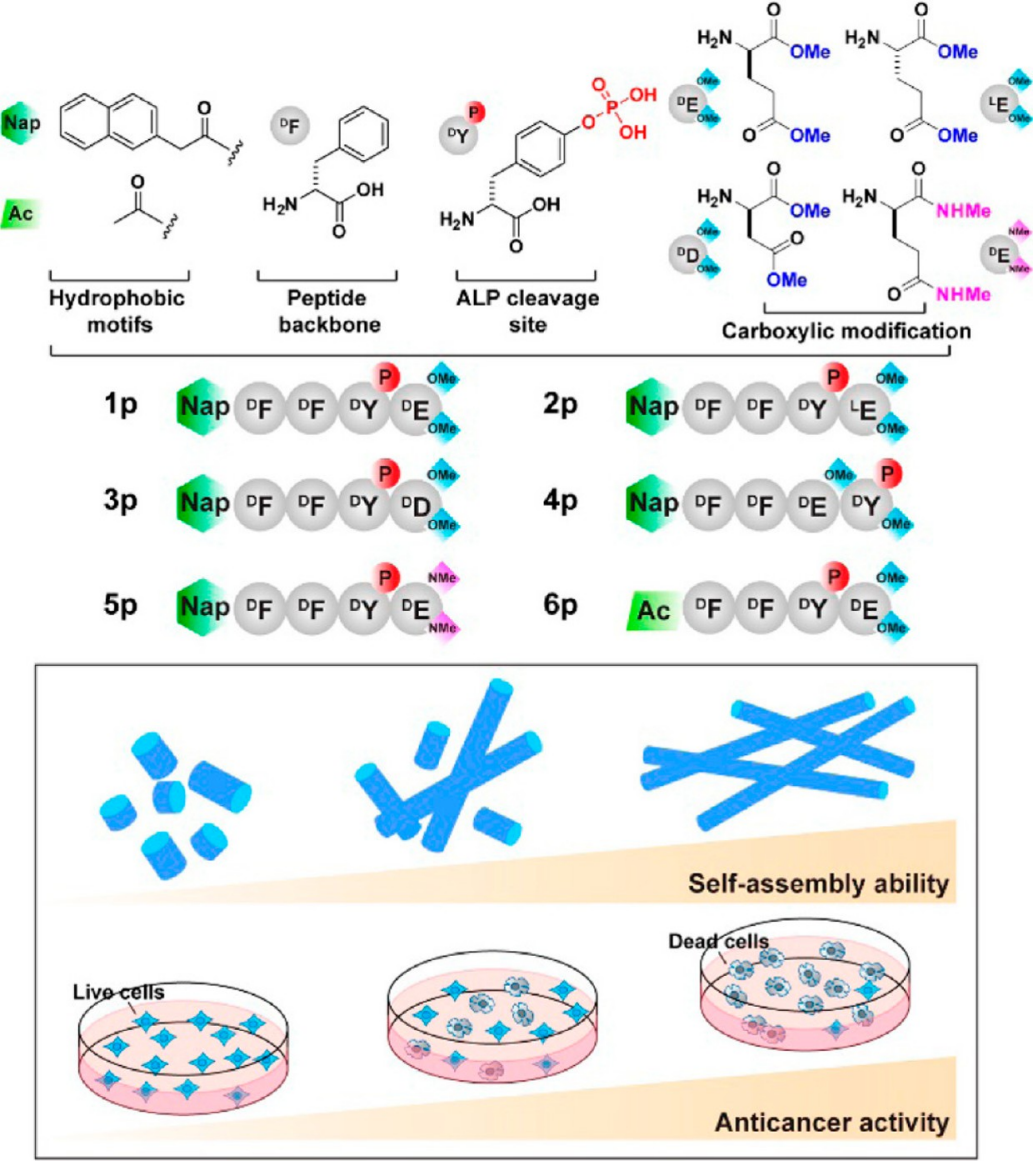
Naphthalene dipeptide derivatives with different C-termini used in a study relating self-assembly ability to anticancer activity. Reproduced from ref (176). Copyright 2017 American Chemical Society.

A competition between assembly and disassembly is observed for the enzyme-driven interaction between Nap-Y-OMe and amino acid amides F-NH_2_, Y-NH_2_ or L-NH_2_.^175^ α-Chymotrypsin drives trans-acylation (forward reaction, leading to temporary formation of a hydrogel of **2** at the top of Figure 16) which occurs under kinetic control. The disassembly reaction due to amide hydrolysis in the presence of thermolysin produces sol-forming **3** (Figure 15). The temporary hydrogelation can be repeated by refueling the system with activated Nap-Y-OMe.^175^

Naphthalene–peptide conjugates have been used to develop targeted delivery agents for cancer therapy. The fact that ALP is overexpressed by cancer cells has been exploited to selectively deliver naphthalene conjugates containing peptides with phosphorylated tyrosine, which upon exposure to native ALP form aggregation-prone peptide conjugates. In an early study, it was demonstrated that anticancer activity is correlated to the propensity to self-assembly after ALP activity, in a series of derivatives shown in Figure 16 with modified C-termini.^176^ The aggregation propensity was quantified as the free energy change of assembly (from the cmc value) and the −log_10_(IC_50_) value was used as a quantitative measure of anticancer activity against Saos-2 osteosarcoma cells.^176^

In related work, the effect of C-terminal modifications on the self-assembly (hydrogelation) of Nap-ff(py) (after ALP dephosphorylation) was probed, along with activity against cancer cells (Saos-2) compared to noncancerous HS-5 cells.^177^ Derivatives with −OMe or NHNH_2_ C-termini had the greatest (and also selective) activity against cancer cells.^177^ Nap-ff(py) was used in a study of the selective inhibition of cancer cells due to differential ALP expression of HeLa cancer and normal HS-5 cells.^178^ The self-assembly of conjugates bearing one or two d-pY residues was compared, the kinetics of ALP-driven assembly being slower for conjugates with two phosphorylated residues. In addition to naphthalene derivatives, fluorescent analogues bearing NBD were also prepared.^178^ Incorporation of d-amino acids leads to resistance against proteolysis and this has been examined for a series of Nap-FFpY derivatives containing d- or l-amino acids which form hydrogels after treatment with ALP, with different extended nanostructures and with varying cytotoxicity (also assessed for the phosphorylated precursors) against HeLa cells as assayed using MTT viability measurements.^179^ Confirmation of the resistance to proteolysis of derivatives containing any one d-residue was provided by measurements of proteinase K digestion. Endogeneous phosphatase activity in HeLa cells was confirmed to produce the aggregation-prone dephosphorylated molecules.^179^ Nap-ff has been linked to C-terminal taurine (2-aminoethanesulfonic acid), producing a hydrogelator under appropriate conditions with distinct fibril morphologies in sol or gel depending on pH, thermal treatment or sonication required to produce a hydrogel.^180^

The hydrogelation of Nap-FFGEY can be switched using a kinase to drive degelation by tyrosine phosphorylation, and alkaline phosphatase can then be used to recover the gel.^181^ The gel comprises fibrillar structures and is biocompatible, as confirmed by MTT tests on HeLa cells. Gelation in vivo was also observed after subcutaneous delivery in mice.^181^

Another class of peptide conjugate was designed to be responsive to carboxyesterase (CES) and were shown to be selectively cytotoxic to cancer cells. These naphthalene-based hybrids comprise d,d-FF or the two mixed d,l-isomers of diphenylalanine with a sulfonic-acid based group at the C-terminus that is a substrate for CES. The molecules aggregate into fibrils inside cells via EISA in the presence of endogenous CES.^182^ Selective activity of a related conjugate is observed for a pair of cancer cell lines that overexpress ALP but which differentially regulate carboxyesterase.^183^ The CES acts upon a terminal -OMe group and this drives disassembly of fibrils formed after ALP dephosphorylation of a terminal pY residue. Other examples of work by the Xu group on cellular sequestration of peptide conjugates which undergo enzyme-instructed self-assembly, with applications in biomedicine have been reviewed.^12,184^

**Figure 17:**
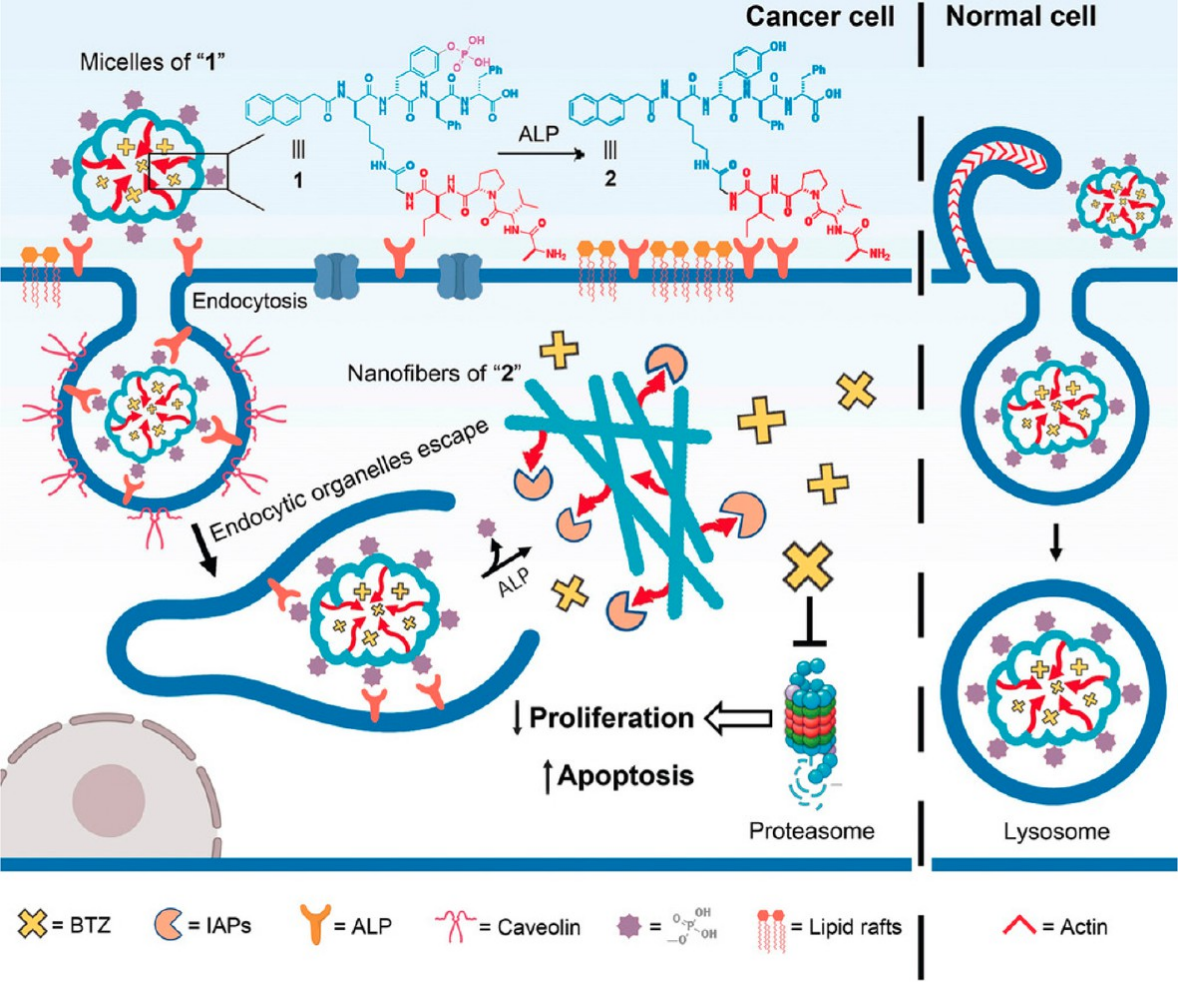
Enzyme-instructed self-assembly leads to aggregation in cancer cells and delivery to the cytosol (and release of micelle encapsulated bortezomib, BTZ), but not in normal cells where lysosomal trafficking occurs. The naphthalene peptide includes appended AVPI, a binding sequence for inhibitors of apoptotic proteins. IAPs: inhibitors of apoptotic proteins. Reproduced with permission from ref (185). Copyright 2020 John Wiley and Sons.

In another example, a peptide motif (AVPI) was appended to a naphthalene peptide conjugate, this being a binding sequence for inhibitors of apoptotic proteins.^185^ As shown in Figure 17, micelles of the conjugate were used to encapsulate bortezomib (BTZ), a clinical proteasome inhibitor that blocks transcription factor NF-κB activation. BTZ is released in the cytosol of cancer cells due to ALP-driven aggregation of the peptide conjugate, but in normal sells BTZ is trafficked into lysosomes.^185^ A naphthalene-based d-peptide conjugate has also been developed that forms fibers due to the activity of phosphatases in the intercellular space, leading to the formation of cell spheroids.^186^ The peptide conjugate bears an appended biotin unit to target cell surfaces. This process mimics the unfolding of fibronectin during remodeling of the extracellular matrix. In a related example, Nap-fff(py)-OMe was used as a substrate for EISA using ALP, including intracellular enzyme and this conjugate was used along with an NF-kB inhibitor BAY to provide synergistic cytotoxicity to cancer cells.^187^ The tetrapeptide conjugate fibrils themselves exhibited little effect on cancer cell viability (although the conjugate lacking C-terminal methylation does).

### 3.3 Other Naphthalene–Peptide Conjugates

**Figure 18:**
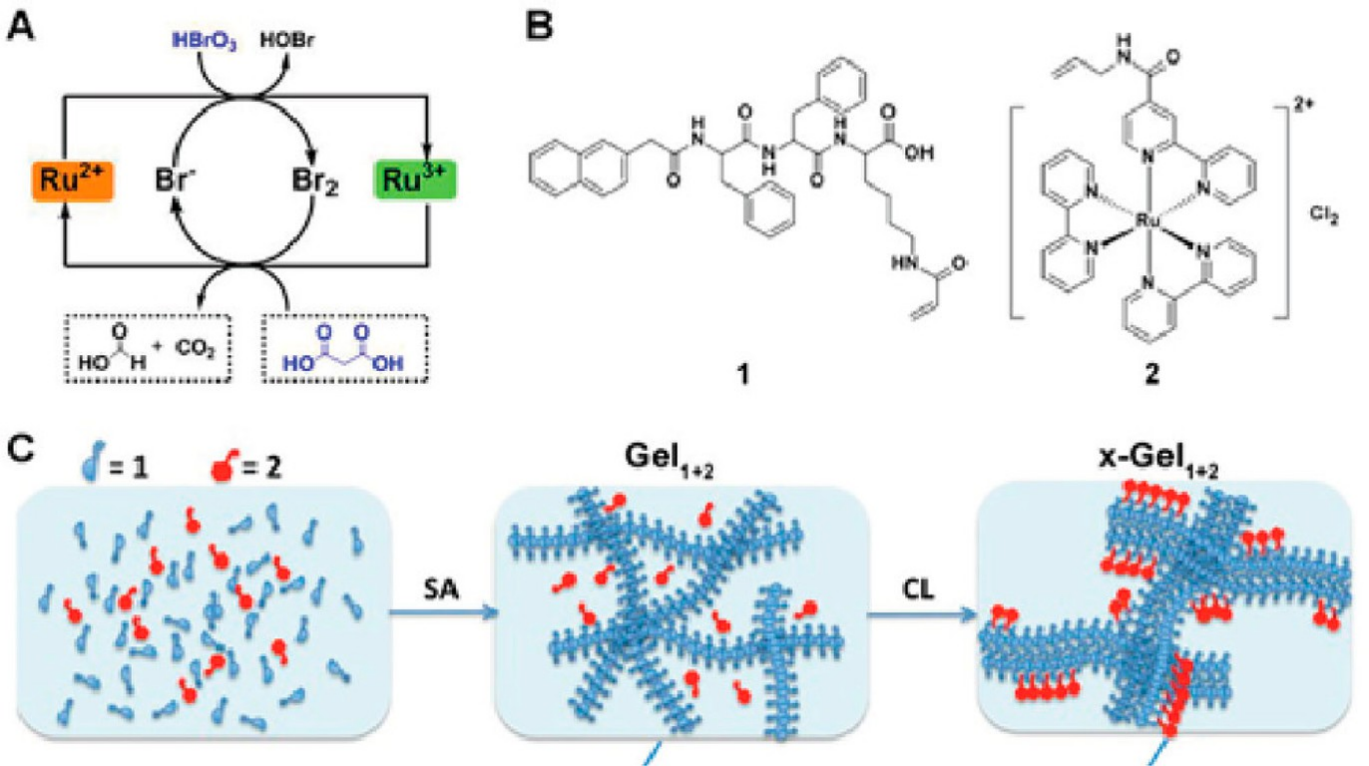
(A) Oscillating chemical reaction (BZ reaction) of malonic acid oxidation by bromate ions catalyzed by a molecule bearing a ruthenium ion complex (as in **2**), (B) Self-assembling Nap-FFK derivative **1** and ruthenium complex **2**. (C) Fibrillar hydrogel formed by mixing **1** and **2** before and after cross-linking.^170^ SA = self-assembly, CL (and x = cross-linking). Reproduced from ref (170). Copyright 2012 American Chemical Society.

A metallohydrogelator has been designed by linking two Nap-FFK molecules via lysine ε-amine groups connected by a pyridine unit. Bipyridine participates in ruthenium(II)tris(byridine) structures, i.e., metal-containing complexes and the Nap-FFK[Ru(bipy)]^2+^KFF-Nap bola-amphiphile forms metal-containing hydrogels.^169^ The photochemical properties of the ruthenium complex lead to UV fluorescence of the hydrogels. Nap-FFK with acrylic acid coupled to the ε-amino lysine (**1**, Figure 18) group was reacted with the molecule **2** bearing a ruthenium ion complex (Figure 18) to produce a hydrogelator bearing a catalyst for the BZ (Belousov–Zhabotinsky) oscillating reaction.^170^ BZ spiral wives were observed in the hydrogel formed after mixing **1** and **2** and cross-linking by UV-induced photopolymerization (**1** and **2** coassemble to form fibrils and hydrogels under suitable pH conditions, and the fibril structure is retained after cross-linking).

Nap-FFK has been used to synthesize a conjugate with nucleotide adenosine monophosphate (AMP) (phosphorylated) via the ε-amino group of lysine.^171^ The Nap-FFK-(phosphorylated)nucleotide conjugate undergoes hydrogelation in the presence of ALP to dephosphorylate the self-assembling molecular precursors. Nucleobases have also been N-terminally attached as aggregation-driving motifs to peptides such as diphenylalanine in trifunctional conjugates with C-terminal glycosides.^172^ These conjugates show good biocompatibility (low cytotoxicity) and facilitate resistance against proteolysis. The conjugate enables the delivery of the nucleobase into the cytosol and nucleus of cells.^172^ In another example, Nap-FFK has been used as scaffold to attach the anti-inflammatory prodrug olsalazine via the lysine ε-amino group.^173^ The diazo group in olsalazine can be reduced (as occurs in the colon, which is targeted by this prodrug) leading to a gel–sol transition and release of anti-inflammatory 5-aminosalicylic acid. A d-amino acid Nap-ffk variant was also prepared and shown to have improved stability against proteolysis.^173^ Other examples of the use of Nap-FF to produce bioactive supramolecular hydrogels have been reviewed.^118^

Remarkably, it has been possible to produced hydrogels containing fibrils of α-helical peptide conjugates, Nap-FFKKFKLKL, with a peptide sequence from associated speck-like protein, associated with the inflammasome.^188^ The peptide is a substrate for plasma pyridoxal-5-phosphate (P5P), the active form of vitamin B6, which has anti-inflammatory activity. Addition of P5P leads to hydrogelation as do other endogeneous molecules including folinic acid and ATP.

Nap-FFYGK modified with a C-terminal cyanuric acid has been used as a detection system for melamine (which has been used to contaminate milk) since cyanuric acid forms a complex with melamine.^189^

Nap-FF and variants with one d-amino phenylalanine or with disaccharide chondrosine (as well as derivatives of NHS, *N*-hydroxysuccinimide including RGD-containing peptides) have been studied as hydrogelators.^190^ The resistance to proteolysis was quantified via proteinase K digestion measurements.

**Figure 19:**
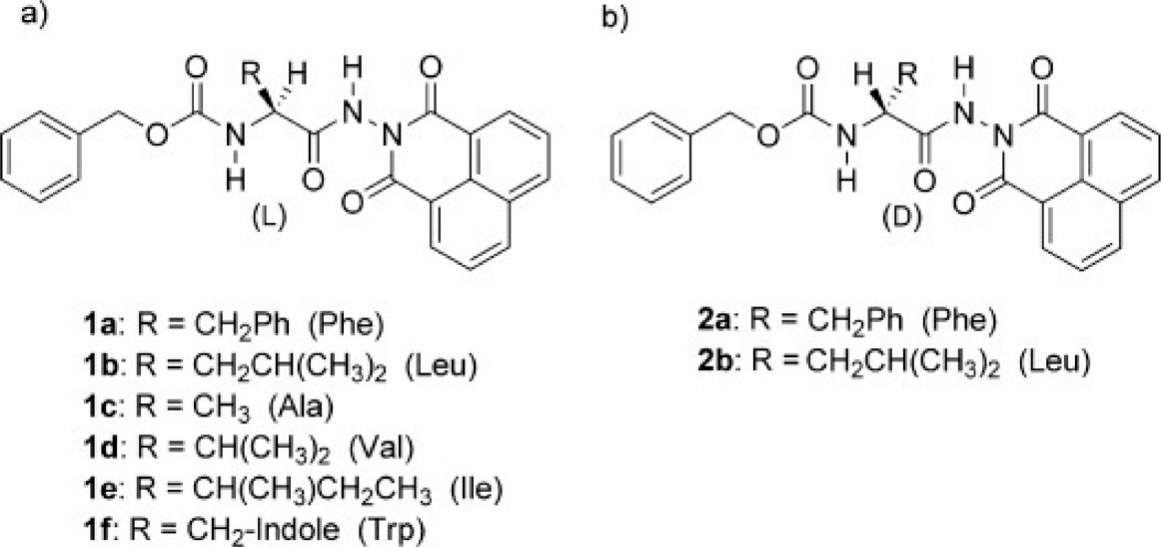
(a,b) Cbz and naphthaloyl-containing molecules, of which **1a**, **1b**, and **2a** gel selected organic solvents. Reproduced with permission from ref (191). Copyright 2011 John Wiley and Sons.

Conjugates with N-terminal Cbz based on F or L with naphthaloyl C-terminal N-protecting group (Figure 19) have organogelation behavior (an analogue with L and N-terminal Fmoc additionally forms organogels).^191^ Organogelation was mainly observed for aromatic solvents, suggesting an important role of π-stacking interactions. Xerogels were found to have a fibrillar structure and the gelation behavior was rationalized based on consideration of Hansen solubility parameters.^191^

## 4 Pyrene

A comparison of the self-assembly of Py-FF with Nap-FF showed a similar fibrillar morphology, although the packing is different.^192^ Py-FF forms aggregates with parallel stacking due to strong π-stacking interactions of the pyrene groups, in contrast to antiparallel stacking of Nap-FF. Also, the mechanism of self-assembly was found to be distinct, occurring via a cooperative mechanism for Py-FF but through an isodesmic process for Nap-FF, based on analysis of variable temperature UV–vis spectra.^192^

Banerjee’s group developed a pyrene-based conjugate Py-FFA-OMe (Py = 1-butyryl acyl) which forms gels in various organic solvent and fluoresces under UV light in *o*-dichlorobenzene.^193^ They also studied the incorporation of graphene nanosheets into the organogel, which substantially enhances the stiffness of the gel.

The stiffness (storage modulus) of hydrogels formed by pyrene linked to di(d-alanine) (aa) via a butyl spacer is boosted by a factor of 10^6^ in mixtures with the antibiotic vancomycin, which acts as a receptor for the pyrene-dipeptide conjugate through hydrogen bonding and π-stacking interactions.^194^ Pyrene conjugates with dialanine form fibrils and hydrogels which exhibit fluorescence. The fluorescence was used to develop sensitive detection systems for nitroaromatic explosive compounds due to changes in the fluorescence intensity of pyrene excimers.^195^ Fibrils of pyrene/aa conjugate are cytotoxic to neurons, however receptor–ligand interactions between the aa dipeptide unit and vancomycin lead to the formation of particles which lack neurotoxicity.^196^

**Figure 20:**
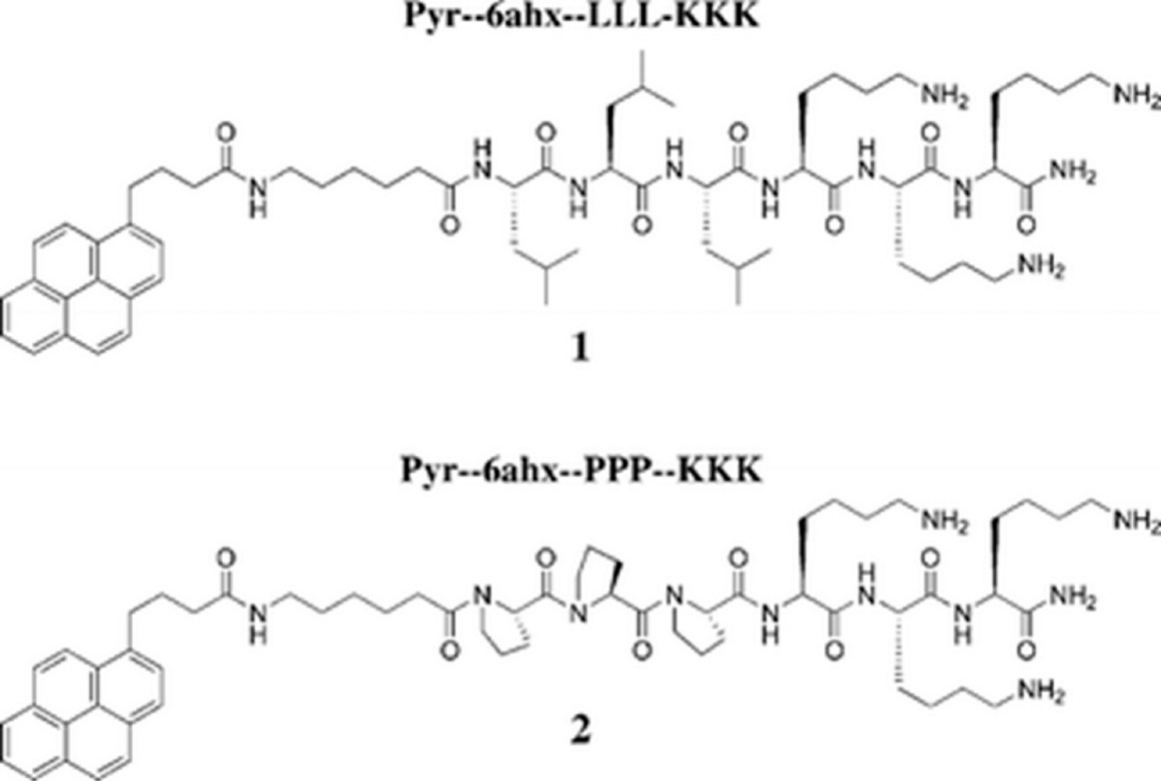
Conjugates of pyrene linked to peptides LLL or KKK via 6-aminohexanoic acid (6AHX). Reproduced with permission from ref (197). Copyright 2005 Royal Society of Chemistry.

Pyrene (in pyrene butyric acid) has been linked via a 6-aminohexanoic acid linker to the N-terminus of a model peptide LLLKKK or PPPKKK (structure shown in Figure 20), where the proline residues in the latter were incorporated to disrupt hydrogen bonding, expected to lead to β-sheet formation for LLLKKK.^197^ Indeed, the former self-assembles into β-sheet fibrils (depending on pH), while the latter forms spherical aggregates with a random coil concentration in acidic and basic conditions.^197^

A conjugate of pyrene (again linked through a butyric acid group) with tryptophan shows hydrogel formation in PBS.^198^ Hybrid gels containing graphene oxide or graphene oxide and gold nanoparticles were also examined. Conjugates of pyrene to tripeptide FFA-OMe form organogels in a variety of solvents.^193^

The coassembly of pyrene conjugates with Fmoc analogues has been examined using spectroscopic methods (CD, FTIR and fluorescence).^199^ Pyr-YL and Fmoc-YL are hydrogelators whereas Pyr-S and Fmoc-S were considered “surfactants”. Pairs of conjugates from this set were studied and orthogonal (Pyr-YL/Fmoc-S or Fmoc-YL/Pyr-S), cooperative (Pyr-YL/Fmoc-YL or Pyr-S/Fmoc-S) and disruptive (Pyr-YL/Pyr-S or Fmoc-YL/Fmoc-S) coassembly processes were identified.^199^

Aromatic stacking interactions of pyrene can be used to drive the β-sheet formation of short peptides extracted from the dimeric interface of a protein (irisin).^200^ The peptides themselves do not self-assemble, but the pyrene conjugates do, as does the mixture (which forms a fibrillar hydrogel). The interacting pyrene units provide conformational restrictions by analogy with the rest of the α-helical protein subunits to which the pentapeptides are pendant.^200^

## 5 Naproxen

**Figure 21:**
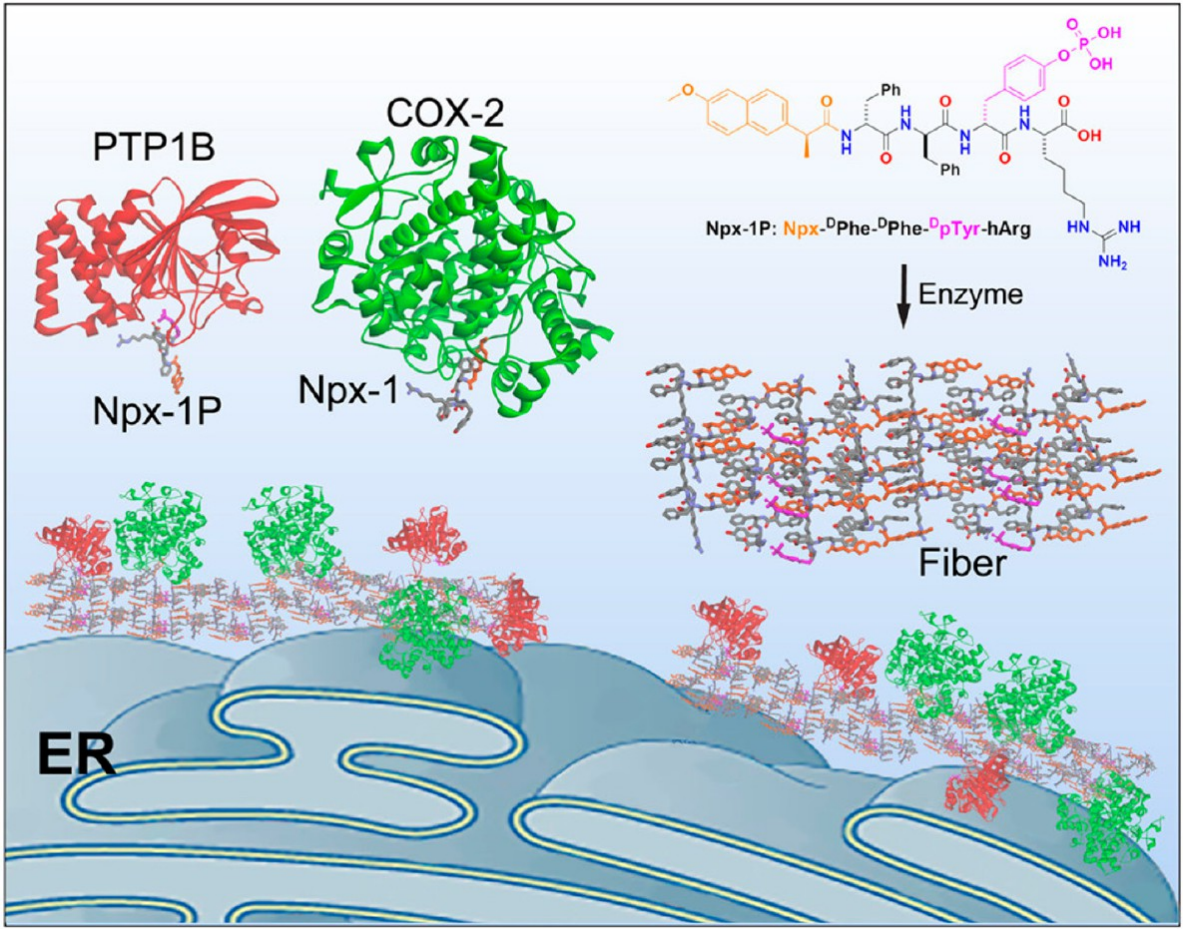
Intracellular assembly of COX-2 and PTP1B on the endoplastic reticulum (ER) via coassembly of fibrils driven by the precursor naproxen conjugate shown (Npx-1P), along with the dephosphorylated molecule Npx-1.Reproduced from ref (202). Copyright 2018 American Chemical Society.

Naproxyl groups (Figure 1) have been used as bulky terminal substituents of peptides and also due to the use of naproxen as a nonsteroidal anti-inflammatory drug (NSAID). Naproxen has been linked to d-amino acid peptides ff, ffy, ffk or ffky as an N-terminal moiety or at the ε-amino group of lysine. Phosphotyrosine versions of the conjugates containing d-tyrosine (y) were also prepared as substrates for phosphatases.^201^ These molecules self-assemble into fibrillar hydrogels in aqueous solution of appropriate pH. They also show selective inhibition of COX-2 compared to COX-1, and sustained release of the hydrogelators from the gels was noted. The highest selectivity was observed for the y-containing conjugates and the d-amino acid hybrids also show better selectivity than the l-residue analogues.^201^ In a later study, a conjugate of naproxen with a ligand of cyclooxygenase-2 (COX-2) was used as a precursor for EISA, achieved via phosphatase treatment of a sequence containing a pY residue.^202^ Co-assembly of dephosphorylated and precursor peptides promoted the coassembly of COX-2 and protein-tyrosine phosphatase 1B (PTP1B) intracellularly at the surface of the endoplastic reticulum (Figure 21).

## 6 Bulky Aromatic Diimides

Naphthalene diimide (NDI) and perylene diimide (PDI) (also known as perylene bisimide) are model bulky π-conjugated molecules with interesting (opto-)electronic properties. Both have been conjugated to peptides. A dipeptide (KK) was functionalized at the ε-amino position of lysine with an NDI chromophore and an N-terminal Fmoc group.^80^ As mentioned in section 2.3, a hydrogel was formed based on peptide conjugate self-assembly into nanotapes and a detailed model for molecular packing in the nanotapes was presented, based on XRD and other methods.^80^ Remarkably, nanotubes were observed for NDI derivatives with lysine as headgroups on each imide unit^203^ or with one lysine headgroup on NDI with a butyl chain on the other imide unit.^204^

**Figure 22:**
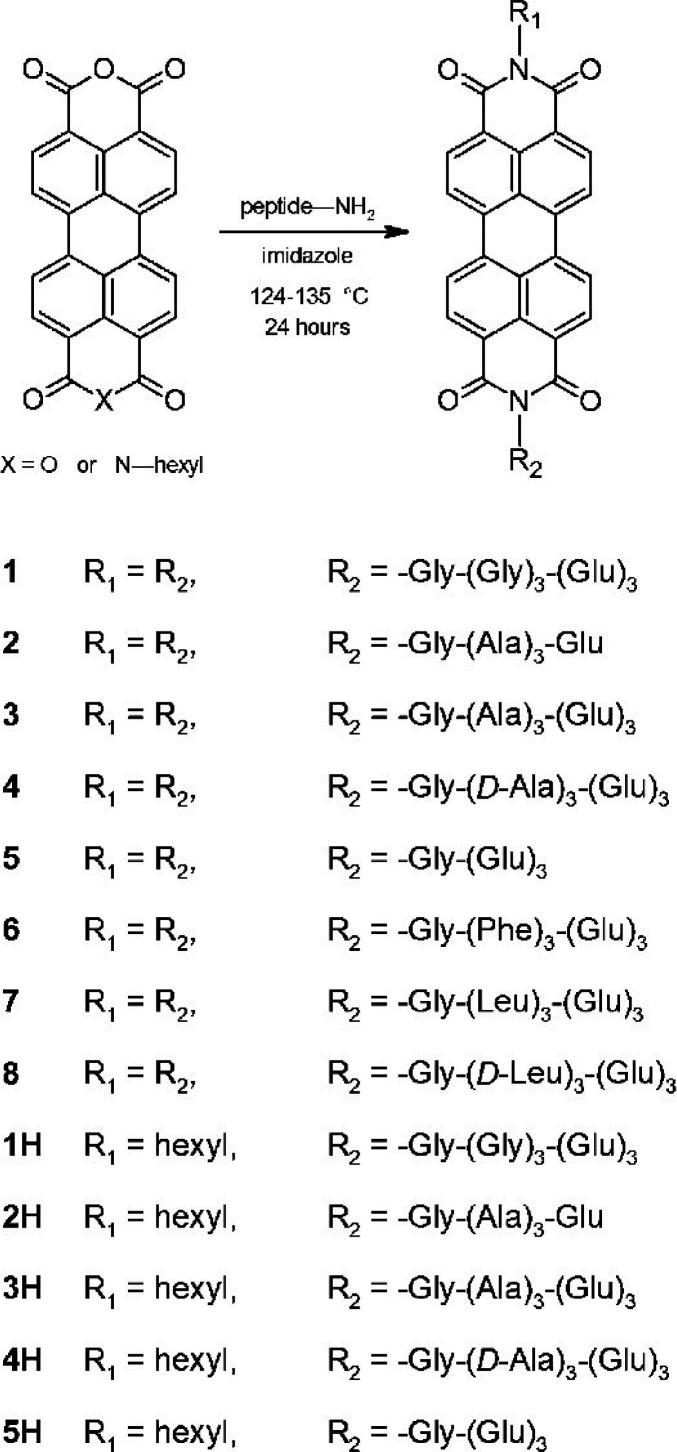
Conjugates of PDI with peptides. Reproduced from ref (206). Copyright 2014 American Chemical Society.

Aggregation of PDI derivatives of bolaamphiphile form with symmetric peptide groups on both imide groups or one peptide and one dodecyl chain was examined using UV–vis spectroscopic methods to probe H- or J-aggregate formation.^205^ In related work, a series of peptide derivatives of PDI with G-X-Y (X = G_3_, A_3_ and others, Y = E or E_3_) tripeptides symmetrically on both imine groups or with a hexyl chain on one imide nitrogen (and tripeptide on the other) (Figure 22) were prepared and self-assembly (fibril formation) was observed for the former class.^206^ Thermodynamic data for all compounds was obtained from temperature-dependent measurements of UV–vis spectra corresponding to H-aggregates.^206^ The self-assembly of two Cbz-l-lysine-functionalized tetrachloroperylene bisimides (4ClPBI-Lys) with α- or ε-Cbz linkage into different nanostructures including nanospheres, fibrils, and nanotapes was observed in acetone/water mixtures.^207^

**Figure 23:**
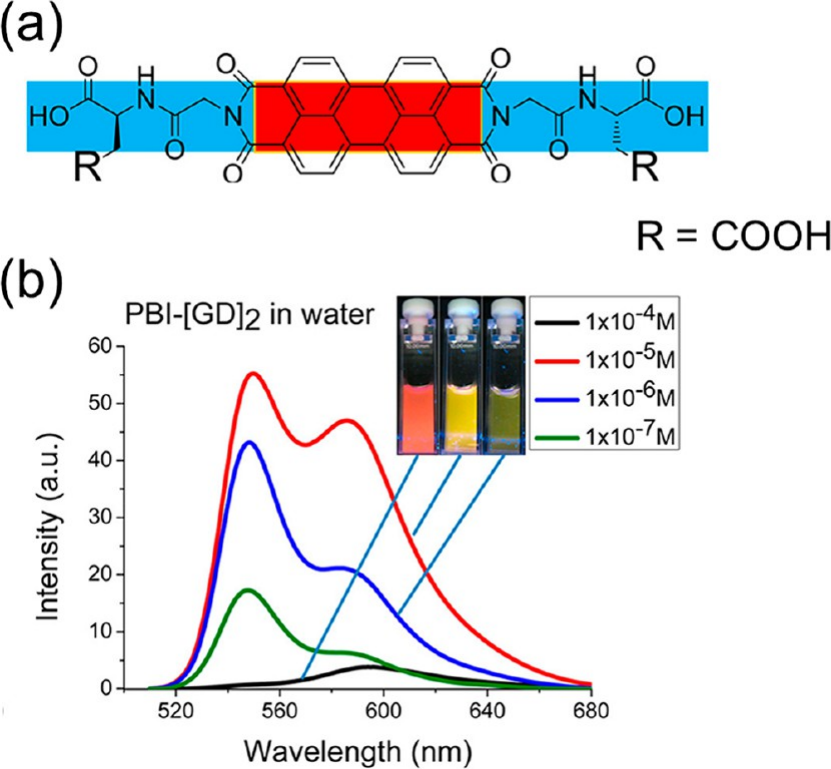
(a) Structure of PBI-(GD)_2_ conjugate (red shading, PBI core; blue shading, dipeptides). (b) Concentration-dependent fluorescence of PBI-(GD)_2_ conjugate in water. Reproduced from ref (208). Copyright 2014 American Chemical Society.

Bola-amphiphiles based on PDI with (GD)_2_ or (GY)_2_ peptide units show fluorescence properties that are tunable in organic solvents (see for example Figure 23), depending also on peptide concentration.^208^ Fibrillar or nanosphere structures were observed depending on the solvent and the (GY)_2_ conjugate also shows gelation behavior in water or DMF, while the (GD)_2_ conjugates can form gels in DMF or DMSO.^208^

**Figure 24:**
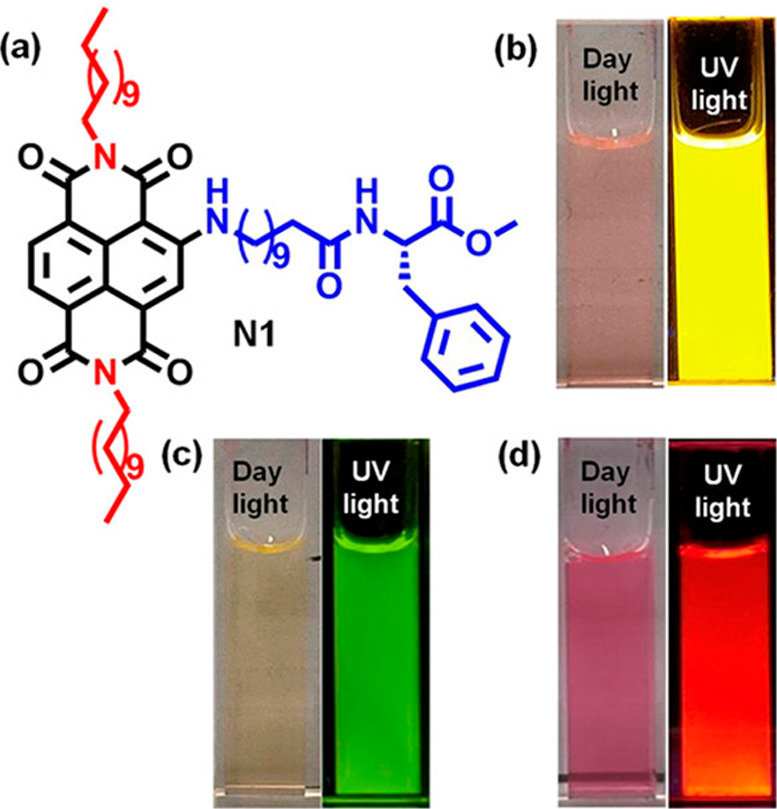
(a) Structure of NDI derivative containing F, images of the conjugate N1 in (b) chloroform, (c) *n*-hexane, and (d) water in the presence of micelle-forming surfactant. In panels (b–d), the left panel shows the sample in daylight, and the right panel shows solutions under UV (365 nm) irradiation. Reproduced from ref (210). Copyright 2021 American Chemical Society.

Bola-amphiphiles have also been prepared with an NDI core, alkyl chain spacers and short terminal sequences of F or V.^209^ These form organogels with a fibrillar structure with J-type aggregates, and undergo aggregation-driven changes in fluorescence, as well as having interesting conductivity behavior.^209^ A core-substituted NDI shown in Figure 24 shows solvatochromism, with different colors in different solvents.^210^ In addition, there is a notable change in fluorescence color in the presence of micelles of cetyltrimethylammonium bromide (due to a change in molecular packing) and finally in water the fluorescence intensity can be used as a sensitive probe of nitrite ions, even in the presence of other anions.^210^

## 7 Other Bulky Terminal Groups

Anthracene has, surprisingly, been relatively less investigated as a bulky N-terminal unit. In one study, hydrogelation in salt solutions or cell culture media was observed for aromatic amino acids F or Y with N-terminal anthracene-2-carbonyl groups.^211^ Photodissociation of the gels was demonstrated due to anthracene dimerization upon exposure to 365 nm light, which was shown to be a suitable means to release cells from the 3D culture environment.

**Figure 25:**
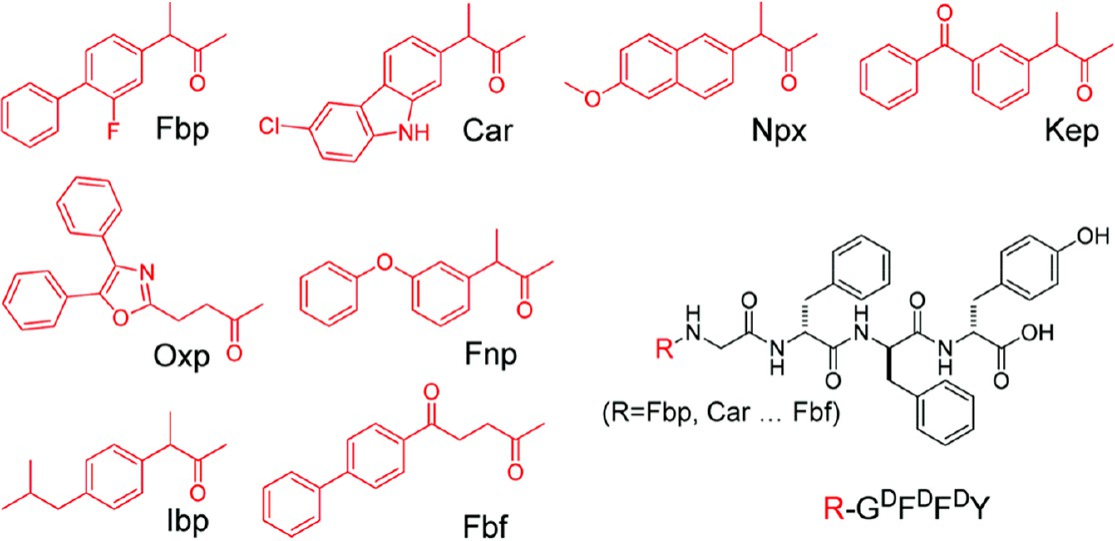
Structures of NSAIDs conjugated to Gffy. Abbreviations: flurbiprofen (Fbp), carprofen (Car), naproxen (Npx), ketoprofen (Kep), oxaprozin (Oxp), fenoprofen (Fnp), ibuprofen (Ibp), and fenbufen (Fbf). Reproduced with permission from ref (212). Copyright 2017 Royal Society of Chemistry.

The Nap-Gffy construct discussed in section 3 has been modified by replacement of Nap with N-terminal NSAID (nonsteroidal anti-inflammatory drug) groups (which are aromatic moieties) including naproxen and ibuprofen (Figure 25 shows structures).^212^ These compounds form fibrillar hydrogels that can be used as vaccine adjuvants by incorporating an ovalbumin antigen in the gel. Increased immunoglobulin production compared to OVA alone was noted, along with enhanced levels of IFN-γ and IL-6 cytokines.^212^

A range of NSAIDs including several of those shown in Figure 25 have been incorporated in self-assembling conjugates to di- and tripeptides including FF, ff, FFY, ffy, and AA.^213^ The hydrogelation properties were examined, fibril structures imaged, and compatibility with HeLa cells tested.

Peptides can be used to direct the self-assembly of phenylenevinylenes. Tovar’s group prepared symmetric bola-amphiphiles with a short (trimeric) oligo-phenylenevinylene core which is π-conjugated and hence has organic electronic properties.^214^ Peptides used to direct structure include those that form amyloid-like fibrils via β-sheet formation such as tetrapeptides AAFD or GAFD.^214^

**Figure 26:**
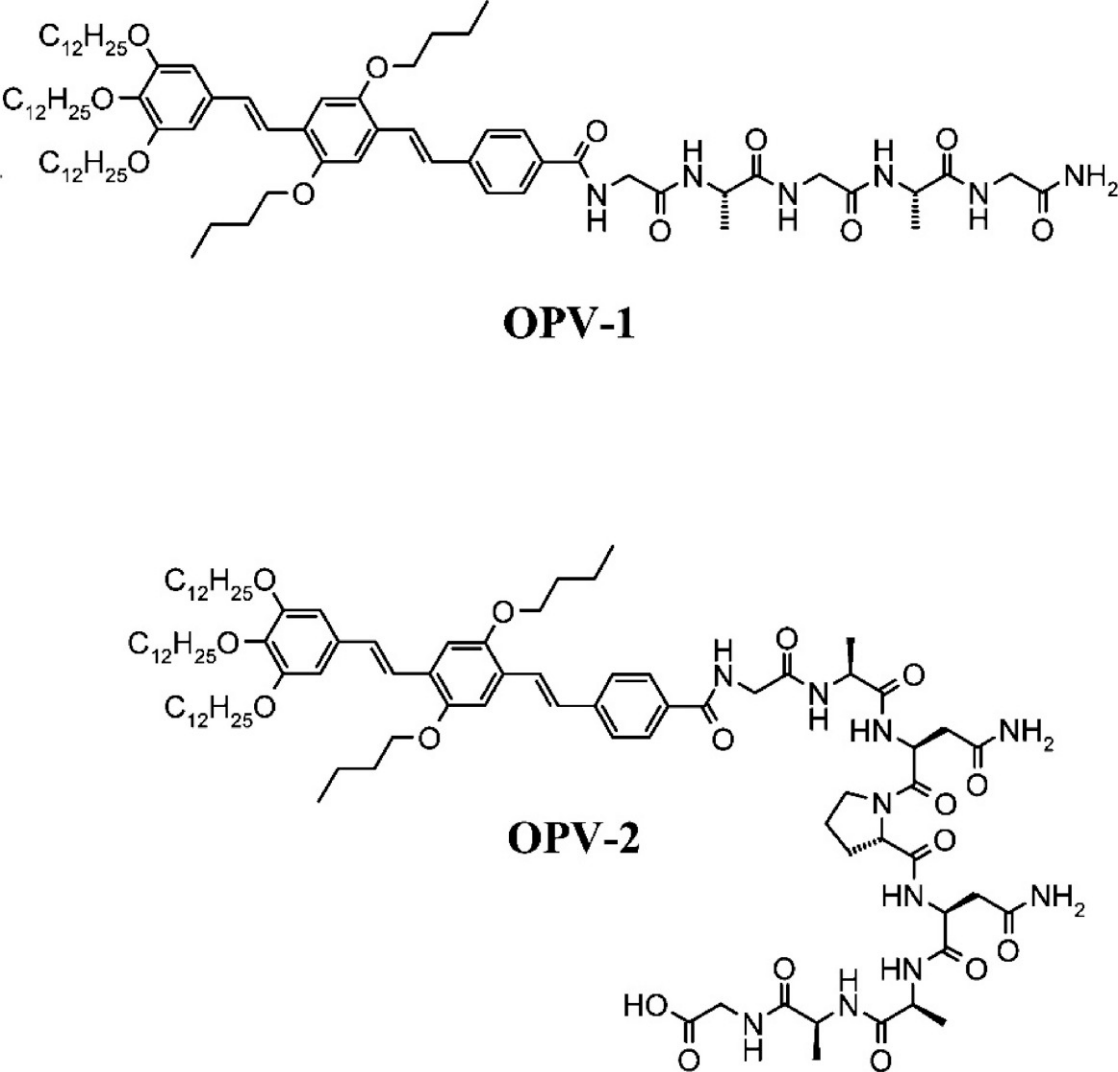
OPV derivatives studied by Matmour et al. Reproduced from ref (215). Copyright 2008 American Chemical Society.

Conjugates of oligophenylenevinylenes, OPVs (Figure 26) with peptides were prepared and their self-assembly examined in aqueous solution.^215^ The peptides were GAGAG or GANPNAAG, the former pentapeptide contains AG repeats observed in crystalline β-sheet domains (in silk). The latter comprises a sequence from CS protein of the malaria parasite, *Plasmodium falciparum*. Scanning tunneling microscopy was possible due to the conductivity of the OPV units, and this showed bilayer arrangements, while in bulk solution, fibrils (cylindrical micelles) were observed.^215^

An electroactive p-type tetrathiafulvalene (TTF)–dipeptide bioconjugate has been created using a TTF moiety appended with diphenylalanine amide.^216^ The hybrid self-assembles into one-dimensional nanofibers that underpin the formation of self-supporting organogels in chloroform and ethyl acetate, in contrast to TTF-L_3_-OMe. Doping of the gels with electron acceptors leads to two-component charge transfer gels in these organic solvents. A π-extended peptide-TTF containing the AGAGA peptide serves as electron-donor in a mixture with a perylene-bisimide (PBI) (nonpeptide) derivative as electron-acceptor, these forming separate *n*- and *p*-conducting nanofibers. Photoconductivity measurements for coassembled systems demonstrated the effectiveness of these heterojunction structures, with applications in optoelectronics and photovoltaics.^217^

**Figure 27:**
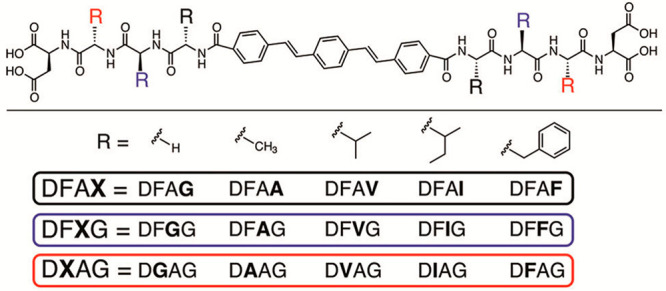
Conjugates of tetrapeptides and 1,4-distyrlbenzene studied by Wall et al. Reproduced from ref (218). Copyright 2014 American Chemical Society.

1,4-Distyrylbenzene has been used as a bulky terminal group in symmetric bolaamphiphiles with terminal tetrapeptides containing D, F, and A or G (Figure 27).^218^ The peptide sequence influences the photophysical properties of the chromophore in the conjugates. Photoluminescence spectra showed features ranging from structured excitonic-like emission expected for H-aggregates to charge transfer-like excimer emission. Fibrils were observed by TEM.

**Figure 28:**
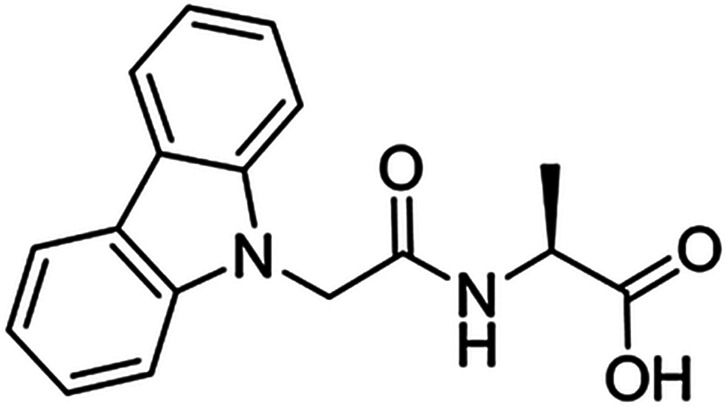
Carbazole–alanine. Reproduced with permission from ref (219). Copyright 2015 Royal Society of Chemistry.

Carbazole–alanine (Figure 28) forms gels via pH switch driven by GdL hydrolysis.^219^ Electropolymerization was also demonstrated to produce microporous hydrogel structures, the resulting films being electrochromic; i.e., it was possible to switch between green (oxidized form) and colorless (reduced form) using cyclic voltammetry.^219^

**Figure 29:**
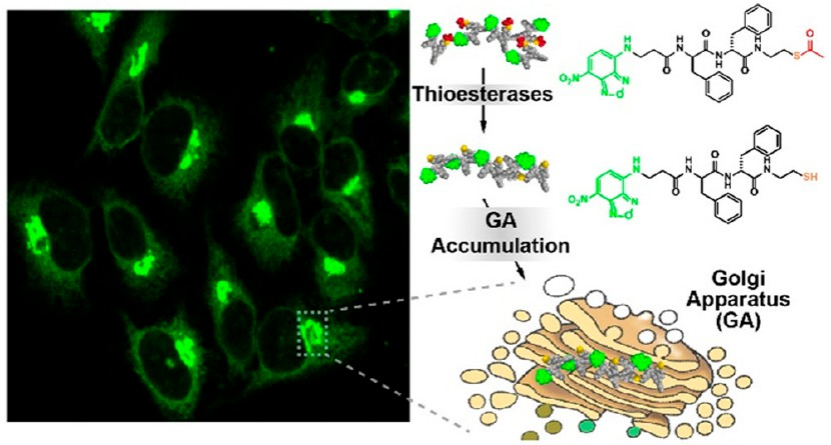
Structures of NBD-ff-thioester and product after exposure to thioesterase in the Golgi apparatus of cells. Reproduced from ref (221). Copyright 2022 American Chemical Society.

NBD has been conjugated to a peptide derived from the second mitochondria-derived activator of caspases and a pY sequence, leading to EISA into fibrils after dephosphorylation (and gels with suitable heat treatment).^150^ NBD-ff has been functionalized with a C-terminal ester cleavage linker to taurine.^220^ The incorporation of taurine was found to greatly enhance the cellular uptake of the d-peptide conjugates, and intracellular esterase activity leads to intracellular fibrillisation. The mechanism of endocytosis was also examined.^220^ NBD-ff has also been used as a fluorophore-bearing proteolysis-resistant scaffold to attach peptide thioesters (Figure 29).^221^ These act as substrates for thioesterases to target the Golgi apparatus (GA) of cells. GA-associated thioesterases hydrolyze the peptide conjugates leading to thiopeptide dimers which accumulate in the GA. This leads to cell death due to disruption of protein trafficking. Thiophosphopeptides such as those shown in Figure 29 can also enter cells via calveolin-mediated endocytosis and undergo dephosphorylation (and disulfide bond formation) to produce thiopeptides that accumulate in the GA.^222^ The thiophosphopeptide also selectively inhibits HeLa cancer cells compared to immortalized noncancerous cell lines.

Attachment of a ferrocenoyl (Fc) group introduces a metallo-aromatic unit into peptide conjugates. In one example, Fc-phenylalanine has been shown to form fibrillar hydrogels (in a pH-responsive fashion), in contrast, Fc-Y, Fc-W, Fc-H, Fc-A and Fc-K do not form hydrogels.^223^ The Fc moiety leads to electrochemical activity, with a potential application as an electron transfer matrix for immobilized enzymes. A conjugate of Fc with the short amyloid-forming peptide VFF forms an organogel in toluene, the fibrillar network structure being disrupted upon oxidation to give spherical micelles.^224^

**Figure 30:**
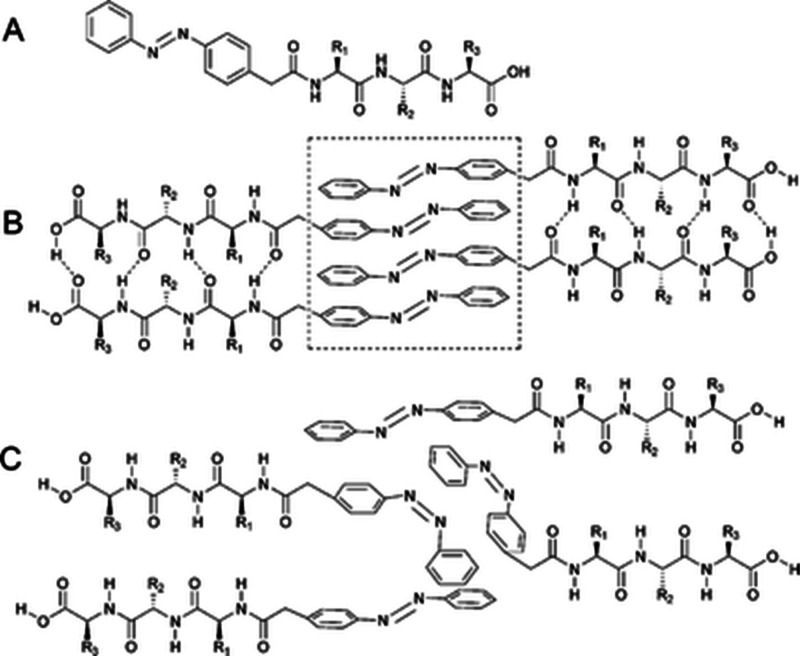
(A) Dipeptide conjugates with N-terminal azobenzene unit. (B,C) Photoinduced conformational switch (*E*–*Z* stereoisomerization) of this unit influences molecular packing. Reproduced with permission from ref (226). Copyright 2011 Royal Society of Chemistry.

Diphenylalanine (with OtBu C-terminus) has been modified with a 4-phenylpyridinium group at the N-terminus to promote cation−π interactions, in a study of dipeptide-based coacervation (liquid–liquid phase separation).^225^

(*E*)-2-(4-(Phenyldiazenyl)phenyl) acetic acid (Figure 30) has been employed as the N-terminal group in conjugates with a series of dipeptides.^226^ The azobenzene moiety can undergo a conformational (stereoisomer) switch in response to UV light, which changes the molecular packing (Figure 30), and this can drive a gel–sol transition. The hydrogelation of azobenzene conjugates with other bioactive peptide sequences such as RGD, and laminin fragments IKVAV and YIGSR were also investigated, and it was shown that the photoresponsiveness could be used for controlled release of the model drug vitamin B12.^226^

Biotin is a bulky unit with bioactivity (it is vitamin B7) that has been used an N-terminal unit in conjugates with peptides such as amyloid-forming sequences (with RGD incorporated in the sequence).^227^ Fibrils are retained for the biotinylated conjugate.^227^

(*S*)-Aroylthiooxime (SATO) has been used as a bulky N-terminal group acting as a thiol-triggered H_2_S donor, in conjugates to the β-sheet IAVEEE hexapeptide with a range of linkers.^228,229^ Fibrils were observed by TEM and gels were formed under physiological pH conditions, from which H_2_S release was quantified.

**Figure 31:**
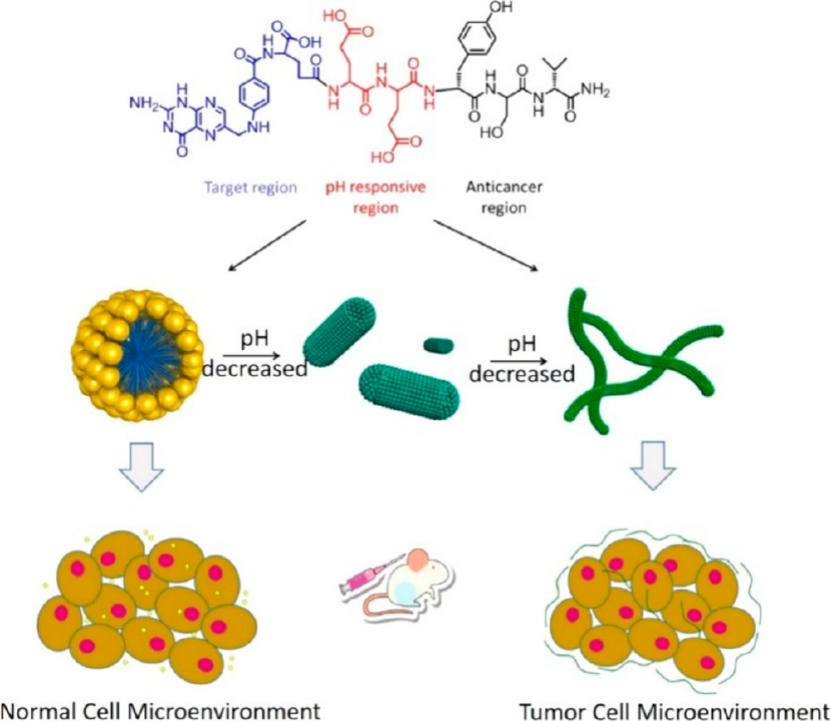
Folic acid–peptide conjugates that show pH-dependent self-assembly and anticancer activity. Reproduced from ref (230). Copyright 2021 American Chemical Society.

Folic acid is of considerable interest in the development of targeted drugs, due to the overexpression of folate receptors in cancer cells.^230^ Conjugates of folic acid with peptide EEYSV (Figure 31) have been shown to form nanoparticles or fibrils depending on pH. The conjugates also demonstrate good selectivity for cancer cells (HeLa compared to NIH-3T3 fibroblasts) and in vivo antitumor activity.

Attachment of the benzyloxycarbonyl (Cbz) unit to FF leads to self-assembly into fibrils, similar to those for Fmoc-FF.^231^ This unit has been used as an N-terminal group for tetrapeptide GFFY, leading to a fibrillar hydrogelator.^166^ Hydrogels with a fibrillar structure have also been observed for Cbz-tetrapeptides with sequences based on arrangements of pairs of F and D residues.^232^ Self-assembly into fibrils is even observed for conjugates of Cbz N-terminally attached to the single amino acid phenylalanine, provided the pH is sufficiently low, and urease can be used to disassemble the fibrils.^233^ In contrast, Cbz conjugated to dipeptides FL, FI, FV, or FY does not form fibrillar hydrogels, although this can be induced by addition of lipase or thermolysin.^234^

Cholesteryl groups were attached N-terminally to peptides A_4_G_3_KRGDS and A_4_G_2_EGRGDS.^197^ These were shown to form fibrils with a β-sheet conformation revealed by circular dichroism spectroscopy under appropriate pH conditions.

## 8 Conclusions and Discussion

This review has highlighted recent exciting work that has explored the self-assembly of conjugates comprising bulky aromatic termini linked to short peptide sequences or even individual amino acids. These bulky units promote self-assembly, primarily through π-stacking interactions. From another perspective, it can be considered that the peptide unit modulates the packing of the aromatic units through peptide hydrogen-bonding interactions (and others), and this can be used to tune optoelectronic properties for example. As highlighted by many examples in this Review, peptide conjugates designed to incorporate bulky aromatic units can be used to create nanomaterials for a diversity of materials science and biomedical applications.

Several major themes emerge from the discussion in this review. First, that the formation of fibrillar hydrogels is a common feature, the fibrils comprising β-sheet structures. The hydrogels have a range of rheological characteristics that cover the stiffness of most tissues in vivo, and beyond. A second feature is the remarkable development of enzyme-responsive self-assembling conjugates using a diversity of enzymes. In one example, this enables intracellular aggregation in response to native enzymes, which can facilitate localization in specific cellular compartments as demonstrated in research by the group of Bing Xu, for example. In another example, reverse hydrolysis can be used to condense peptide conjugates and amino acids, and combined hydrolysis/reverse hydrolysis can occur under thermodynamic control. This type of enzyme-controlled reaction can be combined with others to create enzymatic cascades. There are many opportunities to extend this work to other biologically relevant or catalytically important enzymatic (cascade) pathways.

A third major theme is the distinctive electronic and optoelectronic behaviors that can be produced by incorporating suitable π-stacking and conductive units into peptide conjugates.

Gel-forming peptide conjugates are sometimes considered to be a type of low molecular weight gelator (LMWG). It will thus be interesting to investigate whether methods developed to predict gelation properties of such molecules are applicable to peptide conjugate gelators, although this mainly concerns organogelation which is not a primary focus of research on peptide-based systems. Due to the complex interplay between intermolecular interactions including π-stacking, hydrophobic, electrostatic and hydrogen bonding, it is hard to envisage a universal de novo predictor for peptide hydrogelation or even aggregation propensity, although this has been examined for all known tripeptide sequences.^235^ Machine learning methods hold great future promise in predicting whether a peptide conjugate can form a hydrogel, as demonstrated by work using several machine learning methods.^236^ The QSPR (quantitative structure–property relationships) analysis modeling involved training on a set of initial peptide conjugate gelators (including Nap- and Fmoc-peptides and other compounds) and nongelators (represented as SMILES strings), and then predicting the gelation ability of a set of test compounds, for which 9/9 were accurately predicted to gel (based on tests using synthesized compounds).^236^ There is also scope to apply cutting-edge simulation techniques to model the aggregation of peptide conjugates, although this requires large scale modeling to capture extended self-assembled structures from relatively large (multiatomic) molecules, so coarse-graining methods are required, as for the modeling of smaller tripeptides.^235^

As well as future developments in modeling, we can look forward to further experimental work with bulky units other than those discussed in this review (which actually constitute a rather small subset of potential motifs) with novel structural and functional properties. Distinct and enhanced materials properties (in catalysis, optoelectronics, sensing, and many others) can be expected from the careful design and exploration of peptide conjugates. New directions also beckon in pushing the boundaries of synthetic biology, using responsive peptide conjugates in vivo to functionalize and actuate biological systems, from the cell and beyond.
